# Identification of Selective Inhibitors of the Potassium Channel Kv1.1–1.2_(3)_ by High-Throughput Virtual Screening and Automated Patch Clamp

**DOI:** 10.1002/cmdc.201100600

**Published:** 2012-03-30

**Authors:** Sören J Wacker, Wiktor Jurkowski, Katie J Simmons, Colin W G Fishwick, A Peter Johnson, David Madge, Erik Lindahl, Jean-Francois Rolland, Bert L de Groot

**Affiliations:** a Max Planck Institute for Biophysical Chemistry, Computational Biomolecular Dynamics Group, Am Fassberg 11, 37077 Göttingen (Germany); b Center for Biomembrane Research, Stockholm University, Svante Arrhenius väg 16C, 10691 Stockholm (Sweden); c University of Leeds, School of Chemistry, Leeds, LS2 9JT (UK); d Xention Limited, Iconix Park, London Road, Pampisford, Cambridge, CB22 3EG (UK); e Department of Theoretical Physics and Swedish e-Science Research Center, KTH Royal Institute of Technology, Stockholm University (Sweden)

**Keywords:** drug design, hERG, KCNA2, Kv1.1–1.2_(3)_, virtual screening

## Abstract

**Abstract:**

Two voltage-dependent potassium channels, Kv1.1 (KCNA1) and Kv1.2 (KCNA2), are found to co-localize at the juxtaparanodal region of axons throughout the nervous system and are known to co-assemble in heteromultimeric channels, most likely in the form of the concatemer Kv1.1–1.2_(3)_. Loss of the myelin sheath, as is observed in multiple sclerosis, uncovers the juxtaparanodal region of nodes of Ranvier in myelinated axons leading to potassium conductance, resulting in loss of nerve conduction. The selective blocking of these Kv channels is therefore a promising approach to restore nerve conduction and function. In the present study, we searched for novel inhibitors of Kv1.1–1.2_(3)_ by combining a virtual screening protocol and electrophysiological measurements on a concatemer Kv1.1–1.2_(3)_ stably expressed in Chinese hamster ovary K1 (CHO-K1) cells. The combined use of four popular virtual screening approaches (eHiTS, FlexX, Glide, and Autodock-Vina) led to the identification of several compounds as potential inhibitors of the Kv1.1–1.2_(3)_ channel. From 89 electrophysiologically evaluated compounds, 14 novel compounds were found to inhibit the current carried by Kv1.1–1.2_(3)_ channels by more than 80 % at 10 μM. Accordingly, the IC_50_ values calculated from concentration–response curve titrations ranged from 0.6 to 6 μM. Two of these compounds exhibited at least 30-fold higher potency in inhibition of Kv1.1–1.2_(3)_ than they showed in inhibition of a set of cardiac ion channels (hERG, Nav1.5, and Cav1.2), resulting in a profile of selectivity and cardiac safety. The results presented herein provide a promising basis for the development of novel selective ion channel inhibitors, with a dramatically lower demand in terms of experimental time, effort, and cost than a sole high-throughput screening approach of large compound libraries.

## Introduction

Potassium channels are necessary for many physiological processes, including cell excitability and secretion mechanisms. They are a diverse protein class with more than 75 different types, which can be split into four families: voltage-dependent (K_v_), calcium-activated (K_Ca_), inward rectifier (K_ir_), and two-pore (K_2P_) potassium channels.^1^, ^2^ Voltage-dependent potassium channels are the largest family with 12 members identified to date (Kv1–12) and further subdivisions within each Kv type. In agreement with their broad expression and importance, their dysfunction accounts for a wide range of human pathologies. For example, in the brain, mutations in KCNA1, the gene encoding Kv1.1, are associated with episodic ataxia of type 1,^3^ whereas dysfunction of Kv1.2 is associated with cerebellar ataxia.^4^

Some of the Kv1.x subtypes form heteromultimers, with the Kv1.1 and Kv1.2 combination being one of the most abundant in both the central and peripheral nervous system (CNS and PNS, respectively).^5^–^7^ More importantly, they have been found to specifically co-localize at myelin-protected juxtaparanodal regions of the nodes of Ranvier of nerve axons,^8^–^11^ where they control axon excitability and ensure saltatory conduction.^12^ Nonetheless, in demyelinating diseases such as multiple sclerosis (MS), these channels are exposed and nerve conduction is impaired.^13^ Recently, 4-aminopyridine (INN: fampridine), a nonselective potassium channel inhibitor, has been approved as the first and only medication to improve the ability to walk in those suffering from MS.^1^, ^2^ Unfortunately, its low potency (Kv1.1 IC_50_: 170 µm; Kv1.2 IC_50_: 230 µM) and poor channel specificity (Kv1.4, Kv4.2) raise issues, particularly in regard to cardiac safety. Therefore, the search for more selective blockers, and the development of proper strategies for the study of drug–channel interactions, are highly desirable from a clinical perspective. Supporting Information table S3 lists activity and references to a few other Kv1.1 and Kv1.2 blockers.

The ratio of subunits forming the Kv1.1–1.2 heteromultimer in the CNS is postulated to be one Kv1.1 for every three Kv1.2 α-subunits, as inferred by sensitivity to specific toxins and biophysical profiles. For pharmacological studies, a concatenated construct for a hKv1.1–1.2_(3)_ tetramer was transfected, stably expressed in Chinese hamster ovary K1 (CHO-K1) cells, and validated against the pre-existing hKv1.1_(4)_ and hKv1.2_(4)_ cell lines by means of whole-cell patch clamping, using classical tools such as tetraethylammonium (TEA) and scorpion toxin (tityustoxin-Kα) (data not shown).

Importantly, potassium channels are among the few channels for which structural insight has been gained through crystallography.^14^, ^15^ Functional potassium channels comprise four polypeptide chains assembled in a structure with a central pore. Each of these four proteins comprises six transmembrane domains, and of these, two transmembrane domains (S5 and S6) contribute to the formation of the central pore. The other transmembrane domains play a role in modulation of the activity of the central pore, for example, through the detection of changes in membrane potential and the translation of such changes to modifications in conductance state. The central pore region consists of a solvated cavity ∼10 Å in diameter and a narrow region that is referred to as the selectivity filter. In studies of KcsA and Kv1.5, the inner cavities were used successfully as target sites in structure-based virtual screening (SBVS).^16^–^18^ Furthermore, modeling and mutagenesis studies confirm that the inner cavity is a binding site for small ionic molecules.^19^–^21^ Also, the extracellular entrance of the selectivity filter has been shown to be a target site for inhibitors of potassium channel KcsA^22^, ^23^ and was successfully used for drug discovery using molecular docking.^16^ Based on these results, we chose to use a combination of blind docking^24^, ^25^ and conventional site-directed molecular docking of known ligands to localize putative inhibitor binding sites for Kv1.1–1.2_(3)_.

Virtual screening has emerged as a key methodology in computer-aided drug design. Molecular docking is a virtual screening technique that scores compounds in a known or predicted binding site. Most docking schemes have been developed to work on well-defined binding pockets such as enzymatic active sites, where docking poses can be compared with specific pharmacophores. How well these programs perform on a putative binding site such as the inner cavity of a potassium channel remained unclear. We tested four popular molecular docking approaches (eHiTS, FlexX, Glide, and Autodock-Vina) for their ability to distinguish between compounds known to be active or inactive against Kv1.1–1.2_(3)_.

A commonly used strategy for the improvement of predictions based on molecular docking is the application of consensus scoring (CS). CS involves combining multiple scores to give a new score with a putatively enhanced discrimination rate between active and inactive compounds. The theory behind CS is that the greater the number of independent algorithms identifying a certain substance as active, the higher the likelihood that the compound is a true positive. Enhancement of the enrichment using CS has been demonstrated in several publications.^26^–^28^

Our CS approach is based on individual scoring function sub-terms. It involves a training step, where the most predictive sub-terms of different scoring functions are determined and used for calculation of a consensus score. Different methods of combination of these sub-terms were evaluated. The consensus scheme was optimized for a single target, that is, the inner cavity of KV1.1–1.2_(3)_. The combination with the highest predicted enrichment was applied in a virtual high-throughput screen containing ∼10 million compounds. A subset of 89 top scoring compounds were purchased and assayed by automated patch clamp to assess the achieved enrichment of the applied CS approach.

The aim of the current study was to benchmark, optimize, and employ molecular docking based techniques for a specific target (Kv1.1–1.2_(3)_) in order to find novel and potent inhibitors, thereby decreasing both experimental effort and cost of research. Moreover, to eliminate any cardiac liability, all identified hits were assayed against cardiac channels known to be involved in the cardiac action potential.

## Results

For the benchmark, an in-house compound library was provided from Xention (http://www.xention.com). This library contains activity data for 2675 active and inactive compounds. Owing to the total lack of pharmacological modulators of the Kv1.1–1.2, we mined Xention’s database which contains thousands of compounds with a known pharmacological profile on channels belonging to the same family, in particular Kv1.3 and Kv1.5. The fraction of active compounds in this library is 32 %.

### Blind docking

Possible binding sites were detected by blind docking of the entire test library using Vina. In the blind docking setup, the whole protein structure was defined as the target site. Taking the fourfold symmetry of the receptor into account, blind docking established five distinct putative binding sites. Two separate sites (MEM_1_ and MEM_2_) were found at the protein–lipid surface of the transmembrane region. Further binding sites were discovered at the intra- (V_int_) and extracellular (V_ext_) surfaces of the voltage sensing domains as well as the inner cavity (CAV). The outer mouth of the selectivity filter was not occupied. The benchmark library was docked to each of these binding sites separately. Additionally, the extracellular site of the selectivity filter was defined as a target site (SF_ext_). Within the inner cavity, we used three different locations and sizes of active sites in order to gain further insight into possible binding modes. First, the whole cavity was defined as the receptor (CAV). Secondly, a narrow target site was defined around the inner entrance of the selectivity filter (SF_cav_). Finally, the region surrounding the intracellular pore entrance was defined as a binding site (CAV_ext_).

**Figure 1:**
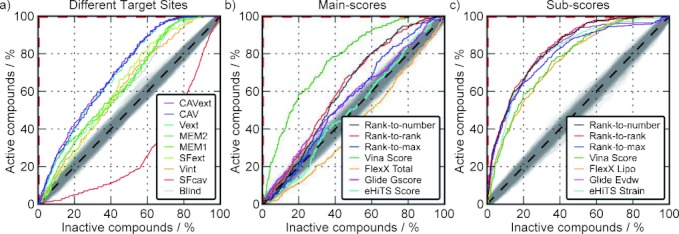
ROC curves a) with respect to different putative binding sites based on Vina Scores; b) ROC curves with respect to the inner cavity of the main scores; and c) scoring function sub-terms, as well as the ROC curves from the respective consensus scores. ROC curves of random distributions are expected in the grey area around the diagonal dashed lines. A perfect ranking corresponds to the red dashed lines.

**Figure 2:**
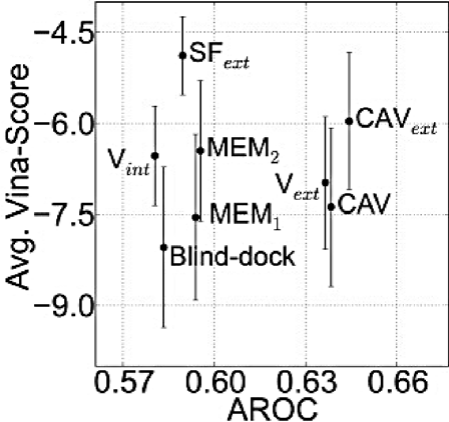
Average Vina Scores over the AROC of different putative binding sites. Error bars reflect the standard deviation of the scores.

The sites defined in blind docking were used as individual target sites. All compounds were docked into the individual binding sites, and the separation between active and inactive compounds was determined using receiver-operating characteristic (ROC) curves. With the exception of the SF_cav_, the rankings for all target sites resulted in significant enrichment of active compounds (Figure 1a). The narrowness of site SF_cav_ led to a systematic exclusion of larger compounds, which explains the low enrichment observed. The highest enrichment was observed for the extracellular pore entry (CAV_ext_), followed by CAV (Figure 1b) and V_ext_. Among the binding sites CAV_,_ CAV_ext_, and V_ext_, the mean scores were lowest for binding site CAV. Lower scores were gained only at transmembrane site MEM_1_ and for the blind docking in total (Figure 2). However, under physiological conditions, the compounds at MEM_1_ would have to compete with lipid molecules at the protein–lipid interface. Therefore, this binding site was discarded, and we focused on the binding site CAV for the subsequent steps.

### Benchmark and optimization

The compound library was further docked to the inner cavity (CAV) using eHiTS, FlexX, and Glide. Of 2675 total compounds, 2099 (including 473 active compounds) were successfully docked by all programs and were therefore included in further analysis. None of these programs enriched active compounds as significantly as Vina. The area under the ROC curve (AROC) was 0.7 for Vina and less than 0.55 for each of the other approaches, represented by the FlexX-Total, eHiTS-Score, and Glide-gscore (Table 1). Furthermore, consensus scoring using the total scores of each program did not provide improved enrichment (Figure 1b). The highest enrichment from consensus scoring using the main scores of all approaches was 0.61 by rank-to-rank. However, taking into account individual scoring function sub-terms from the different programs, a broad range of enrichment was revealed. The sub-terms leading to the highest AROC values for the individual programs were the Lipo term (0.70) for FlexX, the Evdw term (0.74) for Glide, and the eHiTS term Strain (0.73) (Table 1). In the case of Vina, it was not possible to check sub-terms as only the final score was provided.

**Table 1:** AROC and BEDROC values with respect to docking scores and individual scoring function terms as well as their Pearson correlation with compound mass.

Score	PCC^[a]^	AROC	BEDROC
Rank-to-rank^[b]^	–	0.76	0.70
Rank-to-number^[b]^	–	0.75	0.69
Rank-to-max^[b]^	–	0.73	0.69
Mass	1.00	0.74	0.52
Vina Score	−0.82	0.70	0.51
FlexX Lipo	−0.77	0.70	0.59
eHiTS Strain	−0.73	0.73	0.58
Glide Evdw	−0.67	0.74	0.68
Glide Emodel	−0.61	0.71	0.58
eHiTS Energy	−0.46	0.61	0.33
Glide Gscore	−0.20	0.54	0.32
eHiTS Score	0.01	0.50	0.17
FlexX Totale	0.12	0.44	0.11

[a] Pearson correlation coefficients between compound mass and docking sub-score. [b] Consensus scores of the sub-scores Vina Score, FlexX Lipo, eHiTS Strain, and Glide Evdw.

The use of consensus scoring methods using the Vina score in combination with the sub-terms Lipo (FlexX), Strain (eHiTS), and Evdw (Glide) led to a slightly enhanced enrichment in terms of AROC, and a significant enhancement of the Boltzmann-enhanced discrimination of the ROC curve (BEDROC) as described by Truchon et al.^29^ (Table 1). The BEDROC metric is more sensitive to changes in initial enrichment as described in the methods section. The corresponding ROC curves are shown in Figure 1c. The consensus approaches increased the AROC value by 2–5 % and the BEDROC value by 17–20 %, with respect to the average AROC/BEDROC values of the individual terms used for the consensus. As indicated by the increase in BEDROC values, all three consensus approaches led to a significant enrichment in the top 8 % of a ranked list of compounds. We found a strong dependence of enrichment on compound mass, indicating a higher activity on average for larger compounds. However, the rankings according to the three shown consensus schemes were superior to all individual scores and sub-terms.

### High-throughput virtual screening and electrophysiology

As described in the methods section, two implementations of the consensus scheme rank-to-max were used in a high-throughput approach, resulting in two compound sets: **A** and **B**. The compounds in set **A** are based on prediction of a consensus score that was generated using Vina, FlexX, and Glide. The set **B** compounds are additionally based on the Strain term from eHiTS and additional filtering according to drug-like properties. A subset of 89 compounds from the top 200 scoring compounds of either set **A** or **B** were purchased, with the final selection based solely on commercial availability.

Electrophysiological measurements confirmed 14 total compounds exhibiting greater than 80 % inhibition of Kv1.1–1.2_(3)_ when tested at a concentration of 10 μM. The fractions of the identified active compounds in set **A** and **B** are 21 % and 11 % (Table 2). The original ranks for the 14 hits, as well as the ranks within the subset of compounds available at Enamine, are provided in the Supporting Information. One active compound was shared between both sets. Assuming a uniform distribution of active compounds within the first 200 compounds of each list, the number of active compounds can be estimated to be between 11 and 39 % for set **A** and between 3.9 and 25 % for set **B**, with a confidence interval of 95 %. IC_50_ values for these 14 compounds lie between 0.58 and 6 μM. The 14 compounds were evaluated against three important cardiac ion channels: Nav1.5, Cav1.2, and hERG. The experiments reveal a pronounced selectivity for Kv1.1–1.2_(3)_ over the cardiac channels (Table 3). Notably, compounds **1** and **2** were at least 30-fold more active toward Kv1.1–1.2_(3)_ over the other channels.

**Table 2:** Number and fraction of active compounds (+80 % inhibition at 10 μM) from implementations A and B.

	Set **A**	Set **B**	Total
Total	16 829	1285	16 829
Screened	33	74	89
Active	7	8	14
Fraction [%]^[a]^	21	11	17

[a] Fraction of active compounds in the screened subset.

### Analysis of novel active compounds

**Figure 3:**
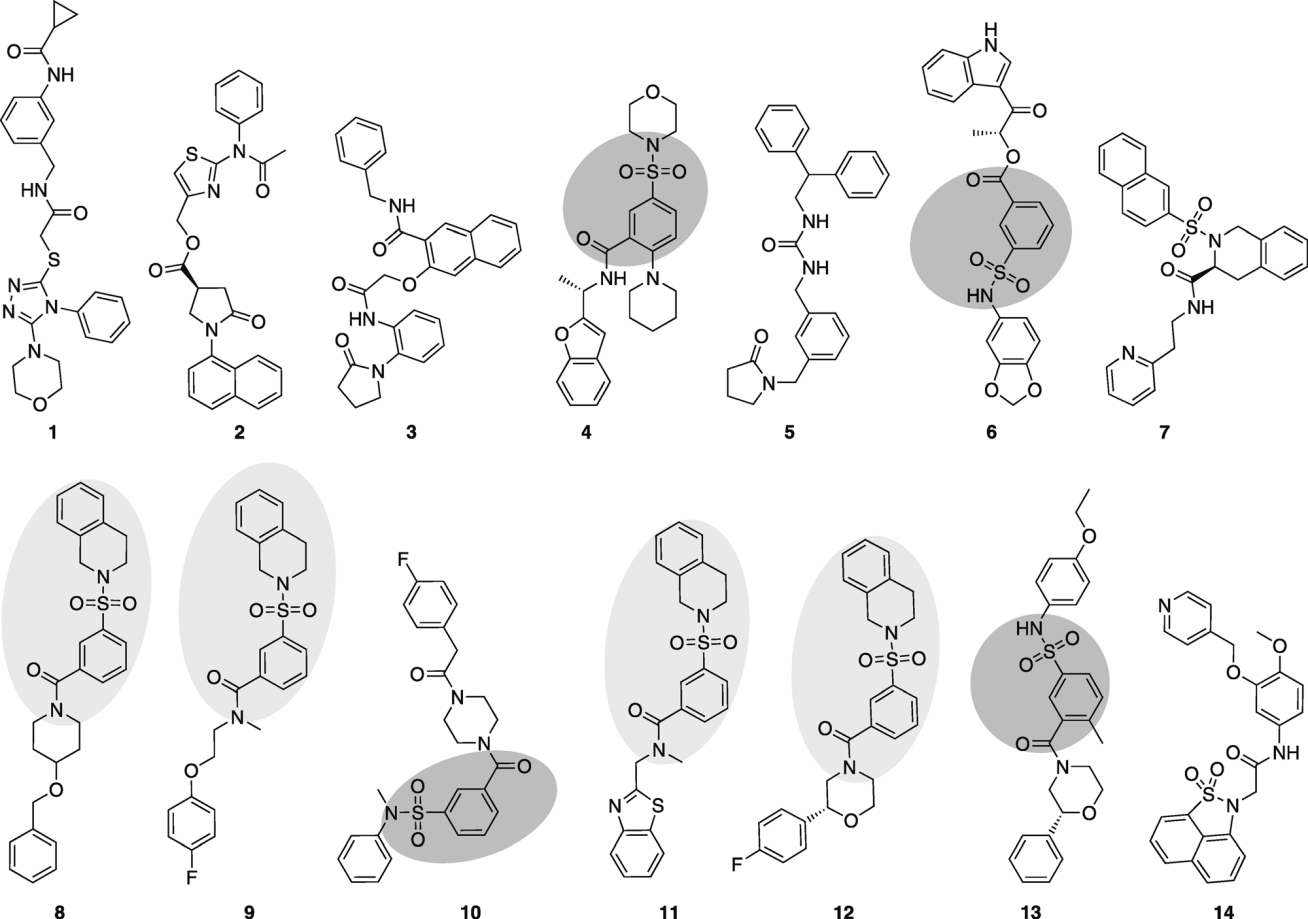
Structures of the 14 confirmed novel Kv1.1–1.2_(3)_ active compounds. Larger repeated motifs are highlighted.

Chemical structures of the 14 active substances are shown in Figure 3. Physiological properties that are relevant for an estimation of their drug-like qualities are listed in Table 3. These data were calculated using the Molsoft drug-likeness and molecular property estimator (http://www.molsoft.com/mprop). The drug-likeness model score predicts drug-like properties using Molsoft’s chemical fingerprints. Values between 0 and 2 indicate very drug-like molecules, although values as low as −1 are frequently reached by drug-like molecules. Non-drug-like molecules usually give values between −3 and −0.5. The distributions of drug-like and non-drug-like molecules are shown on the Molsoft website.^2^

**Table 3:** Molecular properties and drug-like scores of the active compounds. IC_50_ values of the 14 active compounds toward Kv1.1–1.2_(3)_ (primary target) and Nav1.5, Cav1.2, and hERG (from the cardiac safety panel).

ID	Set^[a]^	W^[b]^ [Da]	A^[c]^	D^[c]^	Log *P*^[d]^	PSA^[e]^ [Å^2^]	Vol^[f]^ [Å^3^]	DL^[g]^	Kv1.1–1.2_(3)_	Nav1.5	Cav1.2	hERG
									AV^[h]^	SD	*n*	AV^[h]^	SD	*n*	AV^[h]^	AV^[h]^	SD	*n*
**1**	A	492	6	2	2.64	84	489	0.63	0.71	0.19	3	27.92	4.17	4	+30	+30	0	3
**2**	A	485	6	0	3.28	65	466	0.18	0.79	0.03	3	+30	0	3	+30	+30	0	3
**3**	A	493	4	2	3.91	73	494	−0.67	1.41	0.24	3	24.07	10.27	3	10.44	13.26	3.97	4
**4**	A	482	6	1	4.34	76	482	0.42	1.62	0.48	4	28.38	2.81	3	3.3	8.05	3.53	5
**5**	A	427	2	2	2.76	53	432	1.00	2.98	0.35	4	25.73	7.40	3	+30	+30	0	4
**6**	A	492	7	2	3.56	105	449	−0.15	4.07	0.23	3	+30	0	3	15.5	+30	0	3
**7**	A,B	471	5	1	4.29	67	437	0.08	1.53	0.37	3	+30	0	3	11.43	+30	0	3
**8**	B	490	5	0	5.57	56	472	0.11	0.58	0.11	3	+30	0	4	2.87	7.33	4.75	3
**9**	B	468	5	0	5.32	57	439	−0.32	0.93	0.48	3	29.39	1.06	3	1.55	8.62	2.74	3
**10**	B	495	4	0	3.89	65	464	0.37	1.66	0.21	4	+30	0	4	2.52	9.46	1.23	3
**11**	B	477	6	0	5.59	60	445	0.27	2.71	0.64	6	22.73	9.22	4	3.85	8.43	1.88	3
**12**	B	480	5	0	5.09	57	445	−0.27	3.70	0.60	4	+30	0	3	4.74	8.93	2.05	3
**13**	B	480	5	1	4.38	74	458	0.40	3.77	1.07	3	24.80	9.00	3	9.61	+30	0	4
**14**	B	475	6	1	2.74	81	448	0.07	5.94	0.67	4	+30	0	4	12.27	+30	0	3

[a] Implementation that suggested the compound. [b] Molecular weight. [c] Number of hydrogen bond acceptors (A) and donors (D). [d] Partition coefficient. [e] Polar surface area. [f] Molecular volume. [g] Molsoft’s drug-likeness model score. [h] Concentration that produces 50 % inhibition of the respective channel Kv1.1–1.2_(3)_, Nav1.5, Cav1.2, and hERG, respective standard deviation (SD), and number of evaluations (*n*). No SD or *n* are given for Cav1.2 results, which were obtained using Flexstation. Molecular properties were calculated with the Molsoft molecular properties calculator.

All 14 compounds share a carboxyl group close to their geometric center. Compounds **8**, **9**, and **12** share a Tanimoto similarity greater than 0.8 and have a common 4-(1,2,3,4-tetrahydroisoquinoline-2-sulfonyl)benzamide motif, which is also seen in compound **11**. The similarity of **11** to the former compounds is 0.7 at maximum. These compounds can be regarded as one structural cluster. A second cluster comprises compounds **4**, **6**, **10**, and **13** which each contain a 3-formylbenzene-1-sulfonamide group. Compounds **1** and **2**, which are highly selective for Kv1.1–1.2_(3)_, are not contained in either of these clusters. Twelve compounds contain a sulfur atom, and in 10 cases this takes the form of a sulfonyl group. The molecular weight of the 14 active compounds lies between 420 and 500 Da. The Tanimoto similarity between the 14 active compounds and the known active compounds from the training set was 0.56 at maximum.

## Discussion and Conclusions

In this study, we sketched and validated a possible virtual screening protocol using molecular docking as the main technique. Four widely used molecular docking approaches have been tested for their ability to find known active inhibitors of Kv1.1–1.2_(3)_. In this study, Autodock-Vina led to the best enrichment. Furthermore, we found that using sub-scores from the scoring functions of the individual molecular docking programs can lead to pronounced enrichments of inhibitor identification, even if no enrichment is gained using the main scoring function. Subsequent analysis indicated that the enrichment can be further enhanced by combining these sub-scores into consensus scores. These results underpin the importance of adjustment of the scoring and ranking procedures in a molecular docking calculation for successful virtual screening calculations. The combination of blind docking with conventional docking calculations, as well as the experimental evaluation of our predictions, support the hypothesis that inhibitors bind within the inner cavity of Kv1.1–1.2_(3)_.

Using an adjusted consensus molecular docking approach, we identified several novel, potent, and selective non-peptide Kv1.1–1.2_(3)_ inhibitors. Compounds **1** and **2** represent potential lead structures for the development of novel compounds that could selectively inhibit the ion flux mediated by Kv1.1–1.2_(3)_ in vivo. Electrophysiological measurements confirmed a hit rate at or above 17 % when the relatively stringent hit criteria of greater than 80 % channel inhibition at 10 μM was applied. Four compounds (**1**, **2**, **8**, and **9**) bind in the sub-micromolar range. Compounds **1** and **2** exhibit at least 30-fold greater inhibition potency toward Kv1.1–1.2_(3)_ than they show against a small panel of cardiac selectivity targets (Nav1.5, Cav1.2, and hERG), therefore meeting some of the basic cardiac safety requirements. Evaluation against other unrelated targets was beyond the scope of this research, but further medicinal chemistry could produce a library of similar compounds to aid in the development of a structure–activity relationship for the most active molecules, **1** and **2**. This library could be used to elucidate key binding features from compounds **1** and **2** to guide the development of a novel series of Kv1.1–1.2_(3)_ inhibitors. Neither compound **1** nor **2** has previously been reported in the literature to have any biological activity. Furthermore, there are no references associated with either of these compounds in SciFinder or Reaxys.

The high specificity, as well as the low similarity of these hit molecules to known active compounds from the training set, indicates that this approach makes proper use of the structural characteristics of Kv1.1–1.2_(3)_ in the resulting selection process for the identification of novel structures. The drug-likeness model scores between −0.7 and 1 indicate that the 14 active compounds bear greater similarity to marketed drugs relative to non-drugs, in agreement with the fact that all compounds originate from the ZINC^30^ clean drug-like subset.

Both implementations **A** and **B** identified a similar number of inhibitors with greater than 80 % inhibition at 10 μM concentration. However, set **B** (74 compounds) was approximately double the size of set **A** (33 compounds). The fraction of active compounds was therefore nearly twice as high in **A** than in **B**. This suggests that the enrichment of active compounds is higher when the consensus scoring is applied in parallel rather than sequentially, corresponding to a situation wherein each molecular docking algorithm is applied to each compound. Although such an extensive screen would require substantially more computational time, this may prove to be the most efficient approach, though the influence of successive filtering according to size and solubility applied after the consensus scoring procedure only in case **B** must be taken into account. Nevertheless, we show here that when the whole library was tested with only one docking program and subsequent consensus scoring was applied to a smaller library of top-ranked compounds, an improvement in enrichment of two to three orders of magnitude was achieved compared to a random selection of compounds.^31^–^34^ Because the consensus approach that we used in this study was trained on a library of known active and inactive compounds, this approach cannot be immediately transferred to other targets. However, it may be a reasonable starting point for ion channels that have structural and functional similarity to Kv1.2. Our optimization only targets the scoring and the ranking stages of molecular docking and does not affect the sampling stage. Further improvement might be possible when all three stages are included in the training process.

Finally, the present study shows that when structural information is available, the combination of in silico screening and automated patch clamp may lead to significant acceleration in the ion channel drug discovery process.

## Experimental Section

### Receptor structure

The crystal structure of Kv1.2 (PDB: 2A79) served as template for modeling of the target structure.^15^ Loops that are not present in the crystal structure were added using the MODELLER software.^35^ For Glide, the protein structure was optimized with MacroModel (OPLS2005 force field), and the Protein Preparation Wizard was used to optimize hydrogen bonding networks of the protein.

### Ligand libraries

Experimental data for 811 active and 1864 inactive compounds was provided by Xention Ltd. The Marvin toolkit was used for drawing and displaying chemical structures. Marvin’s calculator plug-ins were used for 3D structure prediction, protonation, and energy minimization.^36^ For Glide docking, libraries were additionally preprocessed by the software LigPrep from the Maestro suite to assign atomic partial charges^37^ and define possible tautomerization states, stereoisomers, and protonation in a pH range of 5.5–8.0.

### FlexX

FlexX^38^ uses an incremental approach for flexible docking of ligands. Initially, the base fragment of a ligand is chosen automatically and placed into the active site. Next, the ligands are incrementally reconstructed. During this reconstruction process, new fragments are fit to the base in all possible conformations. The best of these placements, as defined by the scoring function, are used for the next reconstruction step. The receptor input files for FlexX were generated using an in-house Python^39^ script defining all atoms of the inner cavity within a cylinder of 10.5 Å around the fourfold symmetry axis as the active site. High-throughput screening was performed using FlexX (version 3.1.4). Standard parameters were used for weights of the scoring function and the number of intermediate solutions for each fragment.

### Glide

Glide 5.5^37^, ^40^, ^41^ performs a gradual guided progression solution space search by an initial rough estimate of the ligand conformation and a subsequent torsionally flexible energy optimization on a non-bonded potential grid based on the OPLS-AA force field. The best candidates, as defined by the scoring function, are further refined by Monte Carlo sampling of the ligand pose. Glide’s scoring function is a combination of empirical and force-field-based terms. Intermolecular interactions were precalculated on a grid representing the extracellular half of the receptor and were centered on selected residues in the binding site in such a way as to enable access to total available space in the inner cavity and include long range interactions up to 20 Å. Receptor flexibility was derived by in place temporary alanine mutations and van der Waals (vdW) radii scaling. The 20 000 ligands selected with FlexX screening were docked with full flexibility on the grid. For each ligand, ten poses were generated and subsequently clustered (RMSD<0.5 Å).

### eHiTS

eHiTS^42^, ^43^ takes individual compounds from a large library and calculates the optimal conformation that each of these ligands can adopt in a targeted protein cavity. The program then calculates a score for each structure according to the geometries of the ligand and the complementarities of “surface points” on the receptor and ligand. Complementary surface points receive a positive score, whereas repulsive surface points receive a penalty score. Additional terms are used in the final scoring function to further reflect all factors involved in binding, such as steric clashes, depth of the cavity, solvation, conformational strain energy of the ligand, intramolecular interactions in the ligand, and entropy loss due to “frozen” rotatable bonds. The approach involves breaking ligands into rigid fragments and their connecting flexible chains and then docking each rigid fragment to every possible place in the cavity.

### Vina

AutoDock Vina (version 1.0.2)—hereafter termed Vina—uses an iterative local search algorithm and several runs starting from random conformations. For the local search, a quasi-Newton method is used. Significant minima are then combined and used for structure refinement and clustering.^44^ Input files were generated using the AutoDock plug-in^45^ for PyMOL.^46^ For blind docking, a cubic box containing the complete Kv1.2 transmembrane domain was used. Ligand clusters were defined manually by visual inspection. For targeted docking, rectangular boxes with edge lengths between 1.2 and 3.4 nm around the center of the individual ligand clusters were used. High-throughput docking was performed using 20 000 compounds from the FlexX calculation with the inner cavity as the target site.

### Combination methods

Molecular docking calculations were performed with all programs individually. Scores were standardized using Z-scores prior to consensus scoring. The following consensus scoring methods were used for the generation of a ranked list

• rank2number: Compounds were ranked according to the mean of scores from the different scoring functions.
• rank2rank: Compound rankings were calculated according to the single scoring functions, then compounds were ranked according to the mean of their ranks.
• rank2max: Compounds were ranked according to the maximum of all scores from the different scoring functions.

### Quality evaluation: AROC and BEDROC

The quality of the predictions was evaluated by comparing the predictions of the individual programs with the experimental data in the benchmark library. The predictions were illustrated using ROC curves and were quantified using the area under the ROC curve (AROC) as well as the Boltzmann-enhanced discrimination of the ROC curve (BEDROC), as described by Truchon et al.^29^ AROC is a commonly used metric to evaluate docking applications. The advantage of the BEDROC metric is that it discriminates between early and late recognition of true positives. In this study, a weighting factor of *α*=20 was used for all evaluations, corresponding to 80 % of the score come from the top 8 % of the list. Both the AROC and the BEDROC metric provide values between 0 and 1.

### High-throughput screening

The inner cavity was considered the most promising target site for a virtual screen, which was applied in two steps. The “clean drug-like” subset of the ZINC database from 2009-11-13, containing 9 497 542 entries, was screened.^30^ Initially, the whole database was docked to the inner cavity of the Kv1.2 model using FlexX. The best 20 000 compounds, according to the Lipo term from FlexX, were evaluated in the other programs as well (Glide, eHiTS, and Vina). The prediction of novel Kv1.1–1.2_(3)_ active compounds was based on two slightly different implementations of the CS approach

A) The top 20 000 structures, according to Lipo were docked with Vina and Glide. The rank-to-max consensus method was applied using FlexX’s Lipo-Score, the Evdw term from Glide, and the predicted binding free energy from Vina. Only compounds commercially available from Enamine Sales (http://www.enamine.net/) were taken into further consideration. The 200 top-ranked compounds, according to the CS scheme rank-to-max, were selected.

B) The library of 20 000 compounds was prefiltered to remove compounds that did not fit the drug-like filter of the OpenEye FILTER software.^47^ From 20 000 molecules, 1906 were retained and screened using eHiTS. Ligand dockings were evaluated using SPROUT (version 6.3) and MAESTRO. The rank-to-max consensus method was applied using the sub-score Strain from eHiTS in addition to sub-scores from FlexX, Glide, and Vina, which were also used for the previous implementation. The top 200 compounds, according to the CS scheme rank-to-max and based on availability from Enamine Sales, were selected.

A combined list of compounds from both **A** and **B** was generated. The list was filtered to remove compounds with log *P* values greater than 4.0, in order to ensure sufficient solubility. A total of 89 compounds were purchased from Enamine Sales. The final library of purchased compounds contained 33 ligands predicted by **A** and 74 compounds predicted by **B**; 18 compounds were common to both **A** and **B**. The final criterion for the acquisition of the 89 compounds was commercial availability.

### Electrophysiology

The Kv1.1–1.2_(3)_ channel concatemer was stably expressed in CHO-K1 cells, characterized using conventional patch clamping technique, and adapted to an automated patch clamp device (QPatch16; Sophion, Denmark).

### Creation of a human Kv1.1–1.2_(3)_ expression plasmid

The DNA sequence for hKv1.1 and hKv1.2, corresponding to GenBank Accession Numbers NM 000217 and NM 004974, respectively, were cloned by polymerase chain reaction (PCR) from human genomic cDNA using the proof reading polymerase Pfu (Stratagene). All genes were cloned into the pcDNA3.1 vector. Kv1.2–pcDNA3.1 was cut with Hind III and Bam HI restriction enzymes and used as the acceptor for the PCR product of Kv1.1–pcDNA with primers 5′-TTT TTA AGC TTG CCA TGA CGG TGA TGT CTG GGA G-3′ and 5′-TTT GGA TCC GAT TTC TAA GGT TGA TCG TCG TCC GAA GTT TAA GGT CTC CTT TTG TGT ATC AAC ATC GGT CAG TAG CTT GC-3′ to create Kv1.1–Kv1.2–pcDNA3.1. Similarly, Kv1.2–pcDNA3.1 was cut with Hind III and Bam HI restriction enzymes and used as the acceptor for the PCR product of Kv1.2–pcDNA with primers 5′-TTT TTA AGC TTG CCA TGA CAG TGG CCA CCG GAG AC-3’ and 5′-TTT TTG GAT CCG ATT TCT AAG GTT GAT CGT CCG AAG TTT AAG GTC TCC TTT TGT GTA CGA CAT CAG TTA ACA TTT TGG-3′ to create Kv1.2–Kv1.2–pcDNA3.1.

The hKv1.1–1.2_(3)_ construct was made by PCR of Kv1.2–pcDNA3.1 with the primers 5′-TAG GCT ATG GAG ACA TGG TTC CGA C-3′ and 5′-TTT TTT AAG CTT AAT CTC GAG TGT GCT TCT ACC AAA ATT CAG AGT TTC TTT CTG CGT GTC GAC ATC AGT TAA CAT TTT GG-3′. This produced a construct containing Age I (5′) and a Hind III (3′) site. The section between these sites was removed and ligated into the Age I (5′)- and Hind III (3′)-cut Kv1.1–Kv1.2–pcDNA3.1. This construct was then ligated with Kv1.2–Kv1.2–pcDNA3.1 to create hKv1.1–1.2_(3)_–pcDNA3.1. Constructs were transformed into *Escherichia coli* XL-10 Gold (Stratagene) cells for sequencing, which confirmed all of the constructs.

### Transfection and dilution cloning of CHO-K1 with hKv1.1–1.2_(3)_

CHO-K1 cells (European Collection of Cell Culture: ECACC) were maintained in F12-Ham supplemented with 2 mM glutamine growth media (Invitrogen) with 10 % HyClone fetalclone II bovine serum (FBS) and incubated at 37 °C in 5 % CO2. Cells were transfected using the cationic lipid reagent Lipofectamine 2000 (Invitrogen), following the manufacturer’s protocols. After transfection, Geneticin (Invitrogen) was introduced and used for positive selection. The dilution cloning method was used where cell suspensions were diluted to a concentration in that only a single cell would occupy one well of a 96-well plate (Greiner Bio One). Single clonal populations were visualized and submitted for cell line validation.

### Cell line validation by electrophysiology

Whole-cell patch clamp electrophysiological recordings were carried out using an EPC-9 amplifier controlled by Pulse software (v8.54, HEKA, Germany). The external bathing solution contained (in mM): 140 NaCl, 2.5 KCl, 2 MgCl_2_, 2 CaCl_2_, 10 HEPES, 10 glucose, 23.5 sucrose, pH 7.4. Patch pipettes were filled with an electrode solution of composition (in mM): 100 K-glucanate, 20 KCl, 1 MgCl_2_, 1 CaCl_2_, 10 HEPES, 11 EGTA, 5 ATP-Na_2_, and 2 glutathione, pH 7.2. All experiments were conducted at room temperature (22–24 °C). Cells were held at a voltage of −80 mV. Half activation voltages were calculated using the peak current achieved by stepping the voltage for a duration of 500 ms from −60 mV in 10 mV increments every 10 s to +110 mV. For pharmacological experiments, cells were subjected to a voltage step to +30 mV for a duration of 500 ms every 15 s.

### Adaptation to the automated patch clamp device

The CHO-Kv1.1–1.2_(3)_ cells were then adapted for an automated patch clamp assay. We used the QPatch16 device (Sophion). Each disposable QPlate contains 16 individual patch clamp positions, allowing up to 16 parallel experiments. Cells were detached from T175 culture flasks with 0.05 % trypsin/EDTA solution and kept in serum-free medium (excel 302) in an onboard stirred “cell hotel”. Before testing, the cells were automatically transferred to a mini-centrifuge, pelleted, resuspended in the external solution as detailed above, and washed before being applied to each well of the Qplate. Gigaseals were formed by gradually increasing the suction and, after a short period of seal stabilization, the whole cell configuration was obtained upon execution of a combined suction/voltage protocol. Cell culture conditions were studied for optimal expression and patchability. The Kv1.1–1.2_(3)_ current biophysic was similar to observed values from manual experiments. Patching success was above average (90 %) for an automated patch clamp platform and overall, the currents were stable for at least 30 min (maximum length of an experiment) with an average amplitude ∼2 nA using the following extracellular solution (in mM): 150 NaCl, 10 KCl, 3 CaCl_2_, 1 MgCl_2_, 10 HEPES (pH 7.4); and intracellular solution (in mM): 20 KF, 90 KCl, 5 Na-ATP, 10 NaCl, 10 EGTA, 1 MgCl_2_, and 10 HEPES (pH 7.2).

In the single point experiments, each test compound was prepared from a 10 mM/100 % DMSO mother solution and applied at a final concentration of 10 μM, whereas adequate dilutions were performed for the four-points IC_50_ experiments (0.3, 1, 3, and 10 μM). Compounds were applied via a four-way pipetting robot through integrated glass-coated microfluidic flow channels. All experiments were performed at room temperature.

All whole-cell recordings were taken using a 500 ms depolarizing voltage step from a holding potential of −80 mV to +30 mV, applied every 15 s. Data were acquired and analyzed using the Sophion Qpatch assay software (Sophion).

### Cardiac safety evaluation

Stable transfected cell lines expressing the cardiac ion channels of interest were used on the Qpatch or on the Flexstation (Molecular Devices) using standard protocols. The former machine was used to measure Nav1.5 or hERG currents, whereas the calcium signal (fluo-4) resulting from the Cav1.2 channel activity was evaluated on the latter. Stock solutions (10 mM in DMSO) were prepared and diluted to reach the final concentrations immediately prior to the experiments.

Briefly, For Nav1.5 and hERG, four- and three-points IC_50_ experiments, respectively, were performed on the QPatch (at least *n*=3). For each completed experiment, a fit of the data points (% of current inhibition) was obtained with the Boltzmann equation, and the calculated values of IC_50_ from each experiment were averaged. For the multi-cell Flexstation experiments, the averaged percent inhibitions of fluorescence at each concentrations (*n*=3) were used to construct the concentration–response curves and to estimate the IC_50_ values.

### Spectral data

Purity verification was provided by Enamine Sales. Experimental conditions were as follows: PMR instrument specifications: Bruker AVANCE DRX 500, Varian UNITYplus 400; LC–MS instrument specifications: Agilent 1100 Series LC–MSD system with diode-array detector. Agilent LC–MSD SL mass spectrometer. All LC–MS data were obtained using positive/negative mode switching. Columns: Zorbax SB-C18 1.8 μm 4.6×15 mm Rapid Resolution cartridge (PN 821975–932); ionization mode: atmospheric pressure chemical ionization (APCI); scan range: *m*/*z* 80–1000.

### Abbreviations

Receiver-operating-characteristic (ROC), area under the ROC curve (AROC), Boltzmann-enhanced discrimination of the ROC curve (BEDROC), consensus scoring (CS), multiple sclerosis (MS), Autodock-Vina (Vina).
