# Supplementary material for: Identification of Selective Inhibitors of the Potassium Channel Kv1.1–1.2(3) by High-Throughput Virtual Screening and Automated Patch Clamp
Source: ChemMedChem. 2012 Mar 30;7(10):1775–83. doi: 10.1002/cmdc.201100600 (PMC3525944; doi:10.1002/cmdc.201100600)
Supplement: Supplementary file 1 [file cmdc0007-1775-SD1.pdf]

CHEMMEDCHEM
Chemistry & Drug Discovery

Supporting Information

© Copyright Wiley-VCH Verlag GmbH & Co. KGaA, 69451 Weinheim, 2012

Identification of Selective Inhibitors of the Potassium Channel Kv1.1–1.2$_{(3)}$ by High-Throughput Virtual Screening and Automated Patch Clamp

Sören J. Wacker,[a] Wiktor Jurkowski,[b] Katie J. Simmons,[c] Colin W. G. Fishwick,[c] A. Peter Johnson,[c] David Madge,[d] Erik Lindahl,[b, e] Jean-Francois Rolland,*[d] and Bert L. de Groot*[a]

cmdc_201100600_sm_miscellaneous_information.pdf

Table S1: Original ranks of the 14 hits according to the implementations A and B and the three used consensus scoring methods, rank2max, rank2number and rank2rank.

ID	Implementation A			Implementation B			IC50
	rank2max	rank2number	rank2rank	rank2max	rank2number	rank2rank	
**1**	94	216	55	n.e.	n.e.	n.e.	0.71
**2**	121	373	225	n.e.	n.e.	n.e.	0.79
**3**	86	575	181	n.e.	n.e.	n.e.	1.41
**4**	170	228	208	n.e.	n.e.	n.e.	1.62
**5**	72	455	132	n.e.	n.e.	n.e.	2.98
**6**	91	410	91	n.e.	n.e.	n.e.	4.07
**7**	80	583	152	8	35	10	1.53
**8**	2399	2084	1137	68	184	115	0.58
**9**	418	1086	333	9	70	23	0.93
**10**	323	471	315	7	23	8	1.66
**11**	1204	782	352	133	88	73	2.71
**12**	3563	1805	1401	137	112	53	3.7
**13**	267	740	444	13	51	30	3.77
**14**	303	754	354	112	78	100	5.94

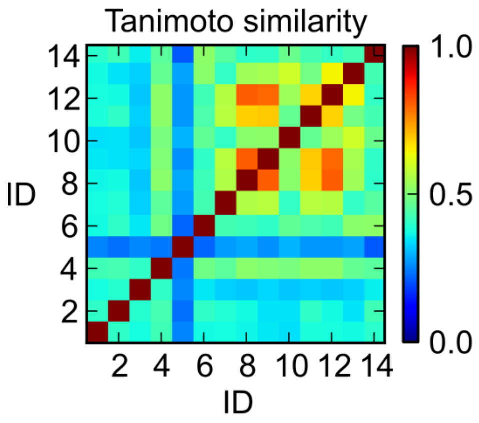

Figure S1. Tanimoto-similarity of the 14 active compounds.

Table S2: BEDROC and AROC values according to scores and subscores of Vina, Glide, eHiTS and FlexX.

Score/Sub-Score	BEDROC	AROC
Autodock-Vina	0.5191	0.706
FlexX_Ambig-Score	0.2356	0.5589
FlexX_AnzMatch	0.1066	0.4086
FlexX_AvgVolume	0.1325	0.5001
FlexX_Clash-Score	0.0422	0.392
FlexX_Lipo-Score	0.5898	0.7029
FlexX_Match-Score	0.0232	0.3718
FlexX_MaxVolume	0.0904	0.4485
FlexX_Rot-Score	0.1874	0.4307
FlexX_Total-Score	0.1094	0.4387
ChemScore_Clash-Score	0.1049	0.4641
ChemScore_FragNo	0.239	0.3545
ChemScore_Lipo-Score	0.5485	0.7312
ChemScore_Match-Score	0.0106	0.327
ChemScore_MaxVolume	0.1068	0.4917
ChemScore_Rot-Score	0.2193	0.4657
ChemScore_Total-Score	0.3361	0.6317
Glide_XP-Electro	0.0256	0.3793
Glide_XP-HBond	0.0215	0.3804
Glide_XP-LipophilicEvdW	0.6058	0.7254
Glide_XP-Penalties	0.1741	0.4052
Glide_XP-PhobicPenal	0.2063	0.5101
Glide_XP-RotPenal	0.0696	0.5022
Glide_XP-Sitemap	0.3578	0.6
Glide_ecoul	0.0259	0.3851
Glide_einternal	0.1101	0.392
Glide_emodel	0.5611	0.7073
Glide_energy	0.5393	0.6945
Glide_evdw	0.67	0.7406
Glide_gscore	0.3146	0.5475
eHiTS_Energy	0.3355	0.6136
eHiTS_Score	0.1648	0.5041
eHiTS_Term-Coulomb	0.1632	0.5416
eHiTS_Term-H_bond	0.0894	0.4321
eHiTS_Term-Lcover	0.1312	0.4798
eHiTS_Term-Lipophil	0.124	0.4542
eHiTS_Term-LlogD	0.0861	0.3868
eHiTS_Term-Rcharge	0.0529	0.3715
eHiTS_Term-Rcover	0.2297	0.4745
eHiTS_Term-RlogD	0.2451	0.5364
eHiTS_Term-Rshape	0.298	0.5632
eHiTS_Term-depth	0.4401	0.6932
eHiTS_Term-entropy	0.0513	0.3396
eHiTS_Term-family	0.4456	0.7025
eHiTS_Term-other	0.2119	0.5413
eHiTS_Term-pi_stack	0.2414	0.533
eHiTS_Term-solvent	0.2521	0.6055
eHiTS_Term-steric	0.3027	0.5495
eHiTS_Term-strain	0.5824	0.7351

**Table S3:** Inhibitors of Kv1.1 and Kv1.2. Sources: Drug Bank, Tocris Bioscience. NA stands for Data Not Available and "-" no indication of particular target					
Name/INN	CAS	Pubmed ID	Kv1.1<br>IC50 (µM)	Kv1.2<br>IC50 (µM)	Other targets/<br>IC50 (µM)
Nifedipine	21829-25-4	7517498	NA	NA	NA
Amitriptyline	50-48-6	17456683	22	-	Kv7.2/7.3<br>10
4-Aminopyridine<br>Fampridine	504-24-5	19413590	170	230	Kv1.4, Kv4.2
Nerispirdine	119229-65-1	19413590	3.6	3.7	NavX<br>11.9
AM 92016 hydrochloride	178894-81-0	15967421	NA	NA	NA
KN-93	139298-40-1	16368898	-	1	Kv1.4, Kv1.5, Kv.21,<br>Kv3.2, Kv4.2<br>hERG

Spectral Data

MaxPeak: 100.00%
Ret_Time: 0.923 min

4532116

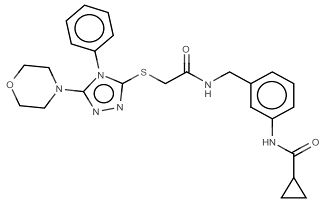

Mol Wt 492.593
Exact Mass 492.22

#	Time	Area%
1	0.923	100.00

RT 0.944

RT 0.944

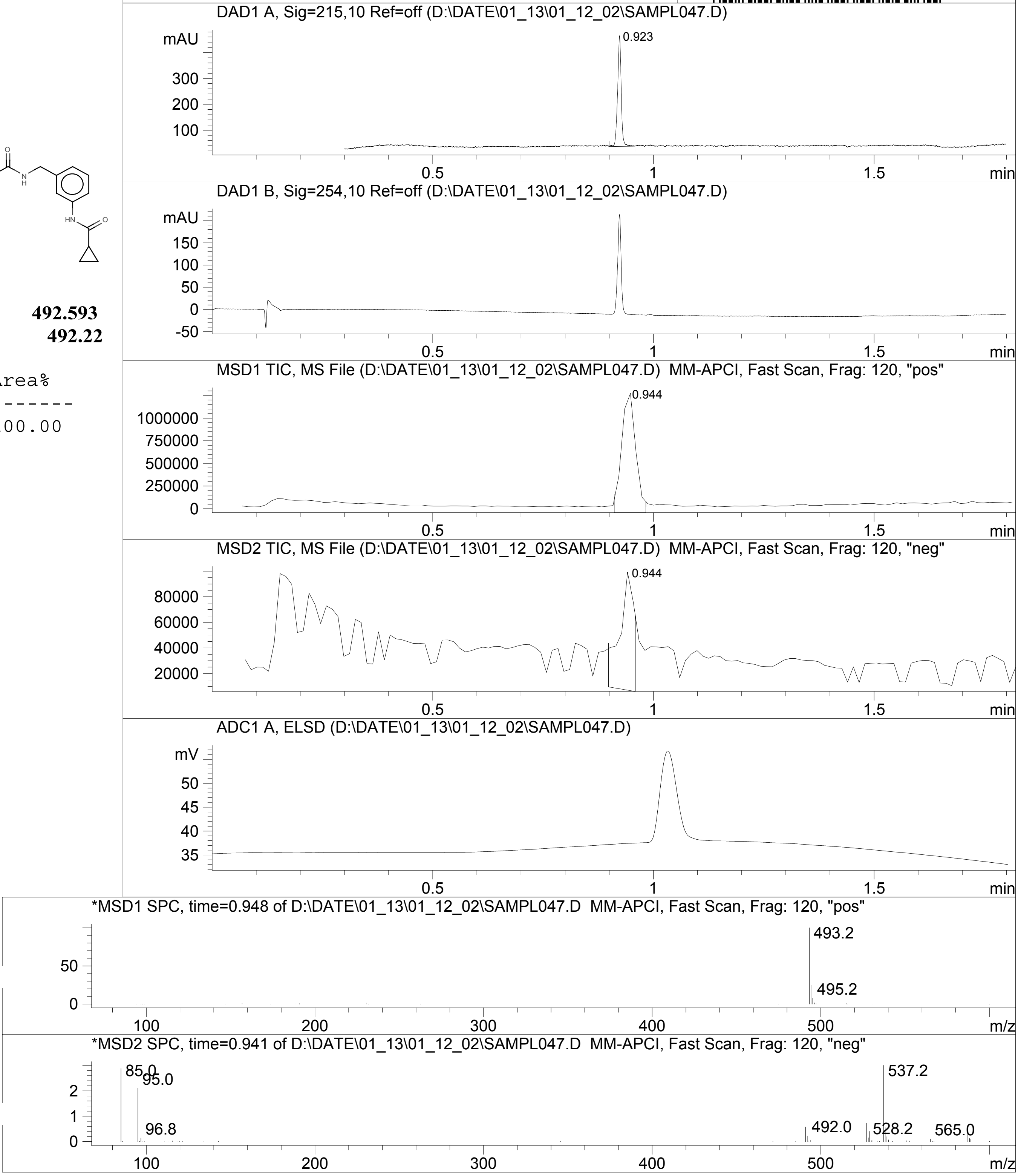

Compound ID: 1

Inj.Date 1/13/2011 L P2-F-02 RR Acq. Method C:\CHEM32\-> ->

MaxPeak: 95.39%
Ret_Time: 1.199 min

4532085

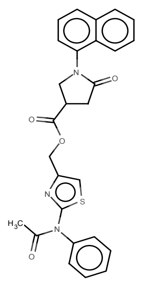

Mol Wt 485.554
Exact Mass 485.16

#	Time	Area%
1	0.844	2.73
2	1.164	1.88
3	1.199	95.39

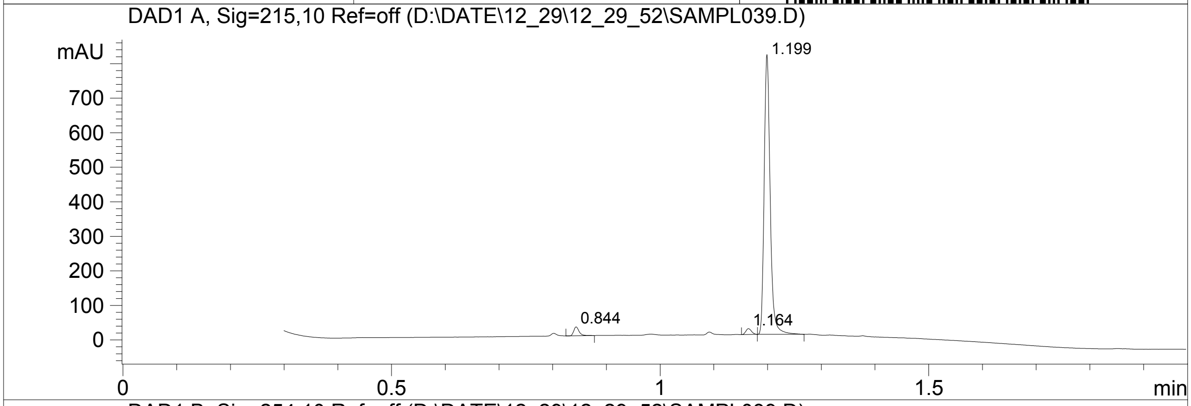

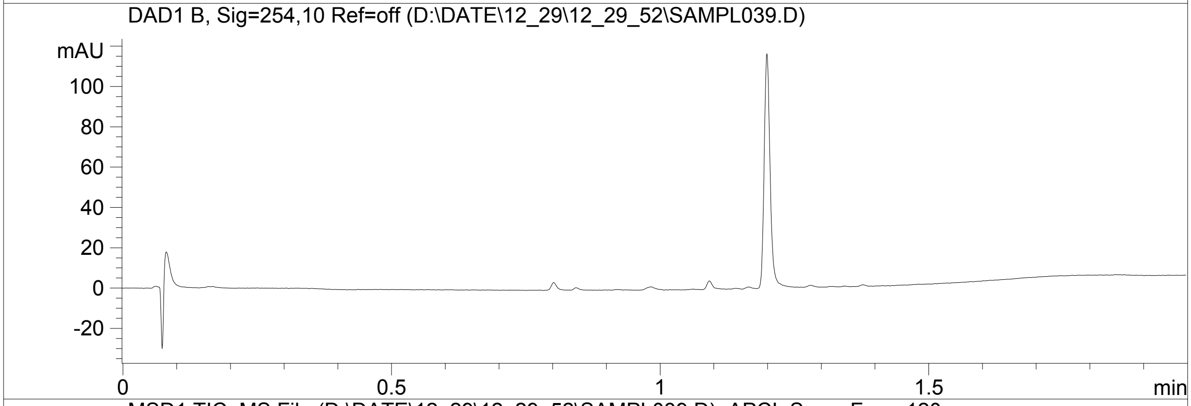

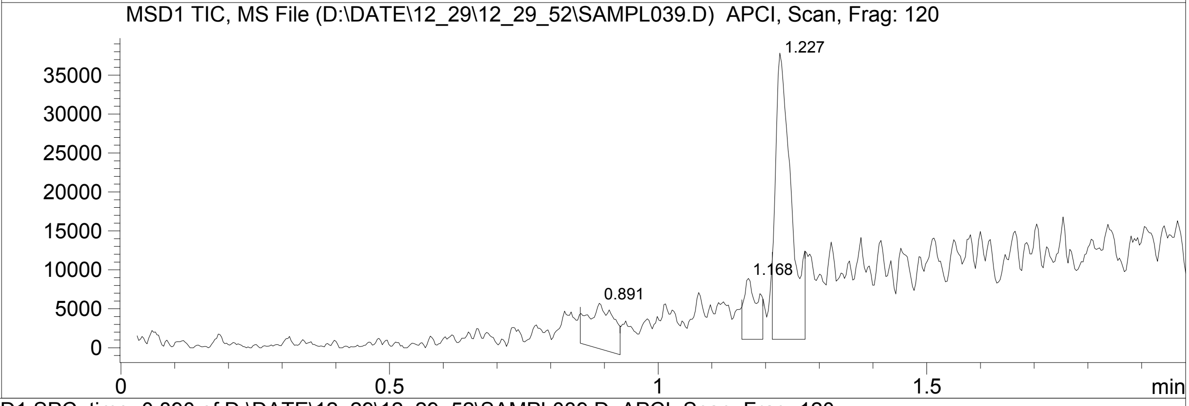

RT 0.891

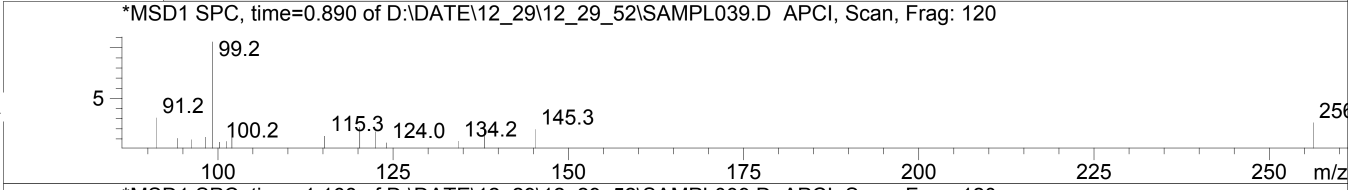

RT 1.168

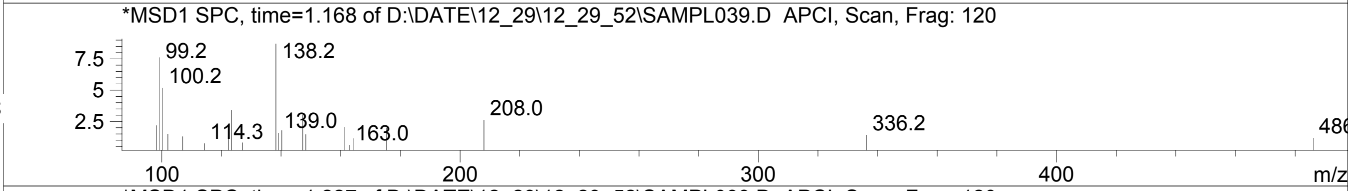

RT 1.227

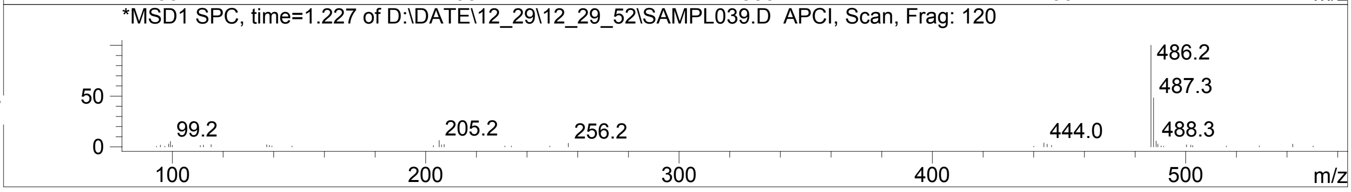

Compound ID: 2

Inj.Date 12/30/2010 O P2-E-02 VL Acq. Method C:\HPCHEM\-> ->

MaxPeak: 94.09%
Ret_Time: 0.740 min

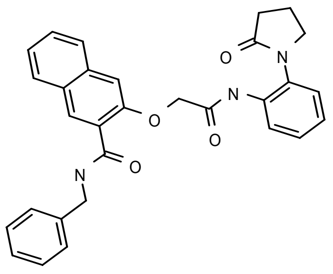

mw = 493.57

#	Time	Area%
1	0.740	94.09
2	0.760	5.91

2013129 OK

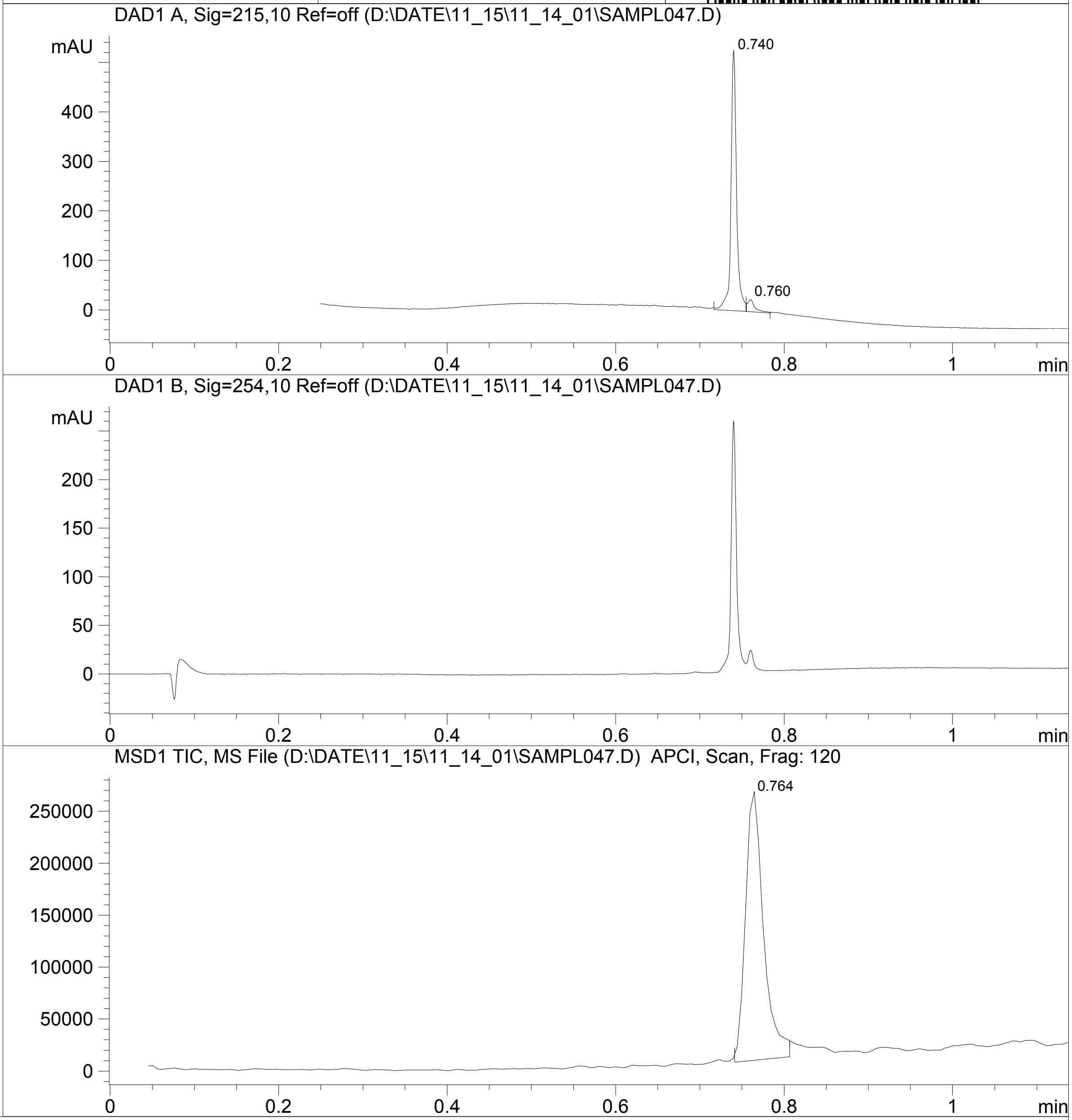

RT 0.764

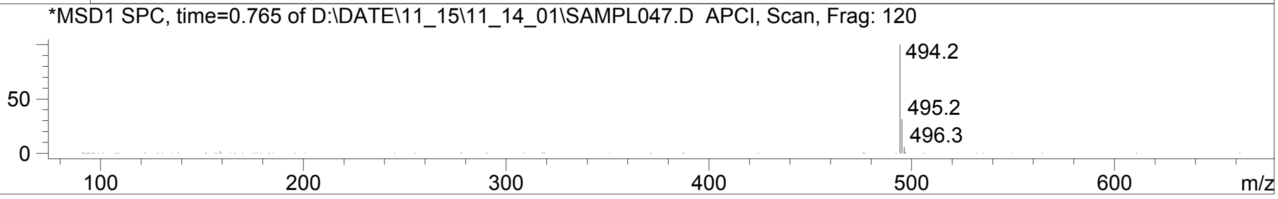

Compound ID: 3

Inj.Date 11/15/2007 E P2-F-03 B03075 Acq. Method C:\HPCHEM\->

MaxPeak: 100.00%
Ret_Time: 1.386 min

4532160

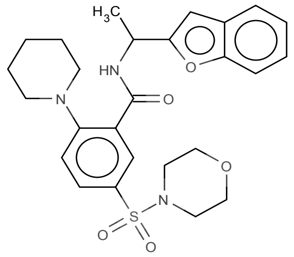

Mol Wt 497.606
Exact Mass 497.23

#	Time	Area%
1	1.386	100.00

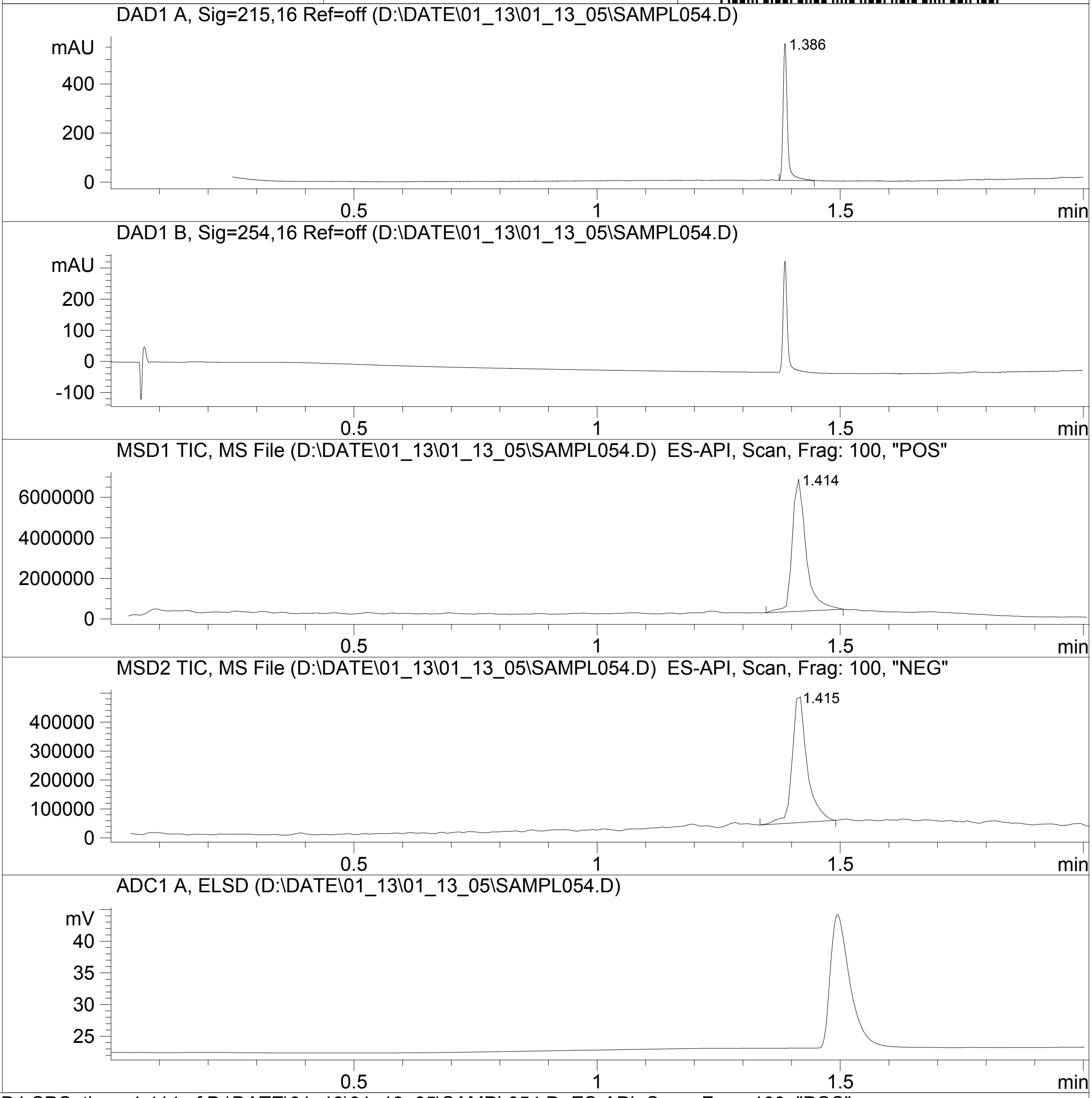

RT 1.414

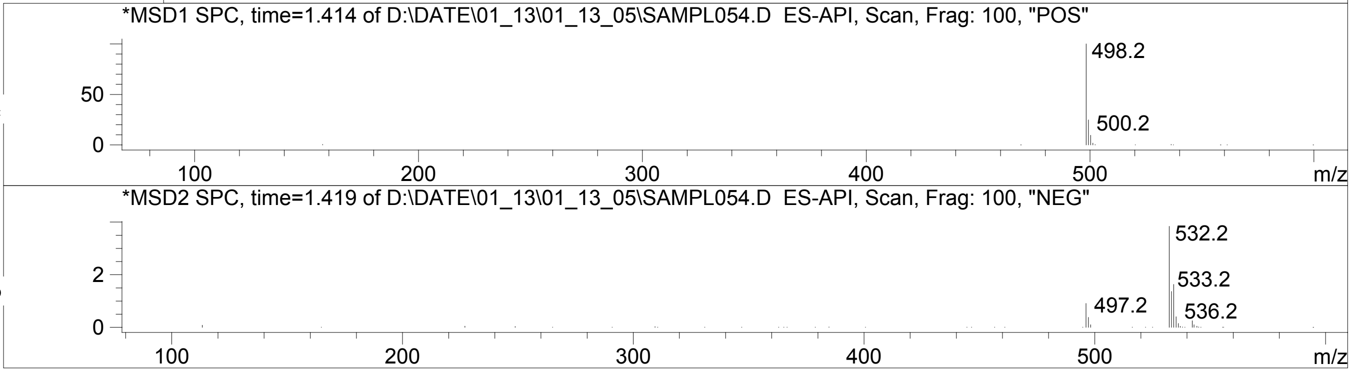

RT 1.415

Compound ID: 4

Inj.Date 1/13/2011 O P2-F-08 '4' Acq. Method C:\CHEM32\-> ->

MaxPeak: 100.00%
Ret_Time: 1.133 min

4531970

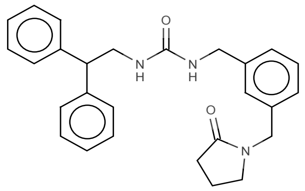

Mol Wt 427.538
Exact Mass 427.27

#	Time	Area%
1	1.133	100.00

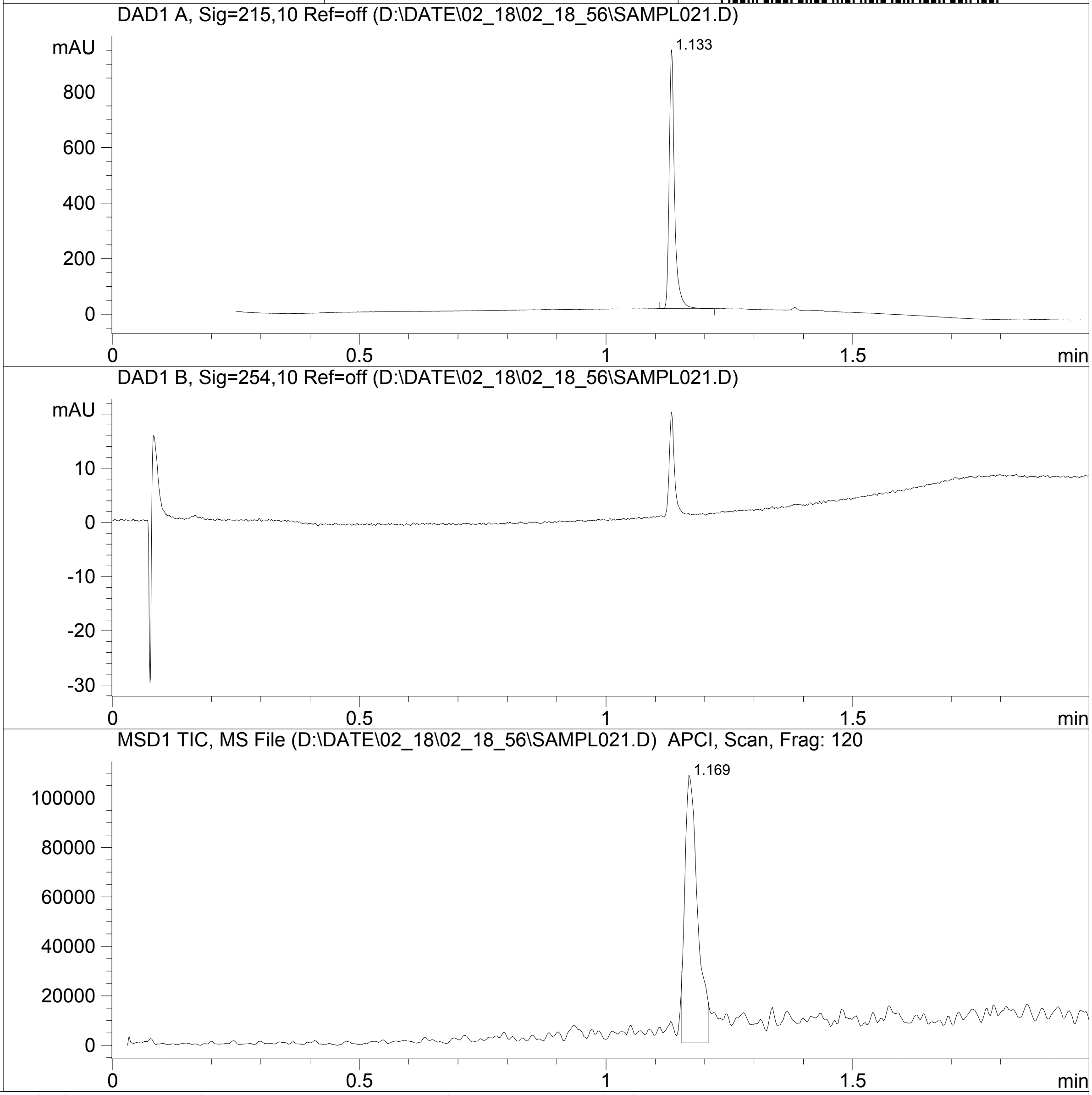

RT 1.169

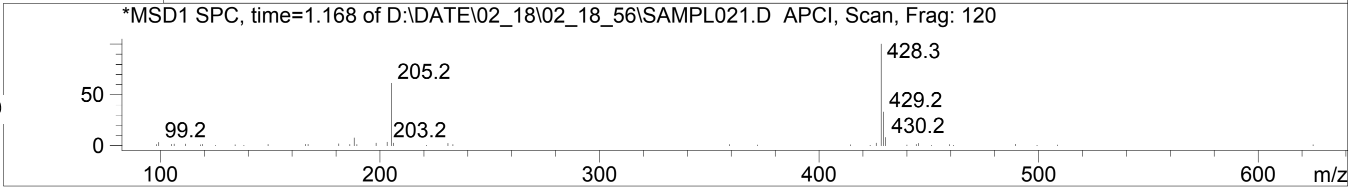

Compound ID: 5

Inj.Date 2/20/2011 O P2-C-02 VL Acq. Method C:\HPCHEM\-> ->

MaxPeak: 91.31%
Ret_Time: 1.165 min

3390363$2

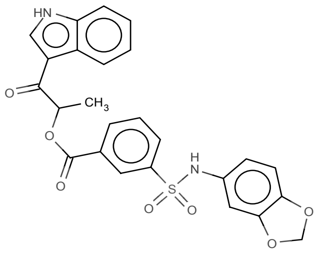

Mol Wt 492.5
Exact Mass 492.1

#	Time	Area%
1	1.165	91.31
2	1.309	2.31
3	1.322	2.91
4	1.389	3.48

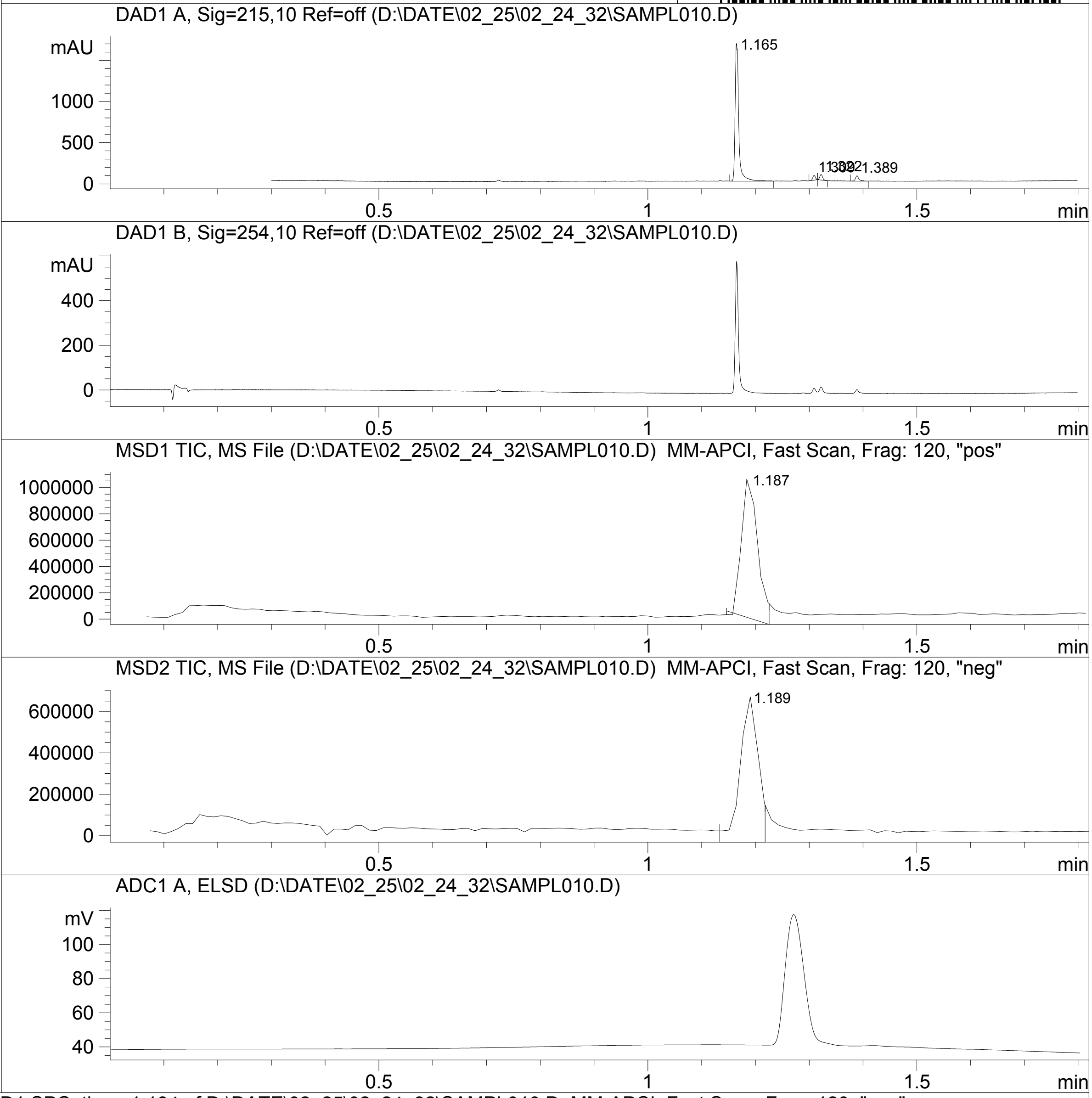

RT 1.187

RT 1.189

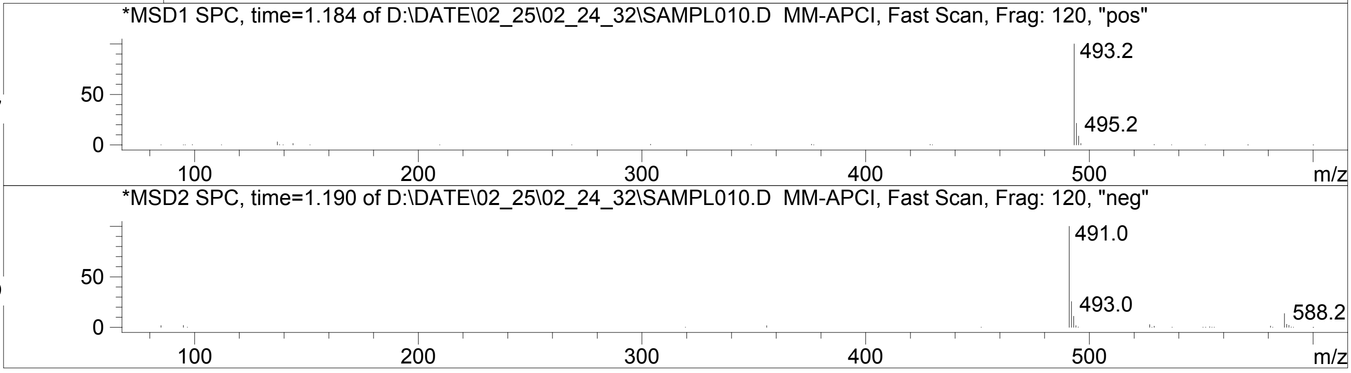

Compound ID: 6

Inj.Date 2/24/2011 L P2-A-09 RR Acq. Method C:\CHEM32\-> ->

MaxPeak: 95.13%
Ret_Time: 0.977 min

4531990

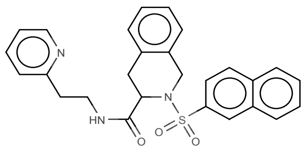

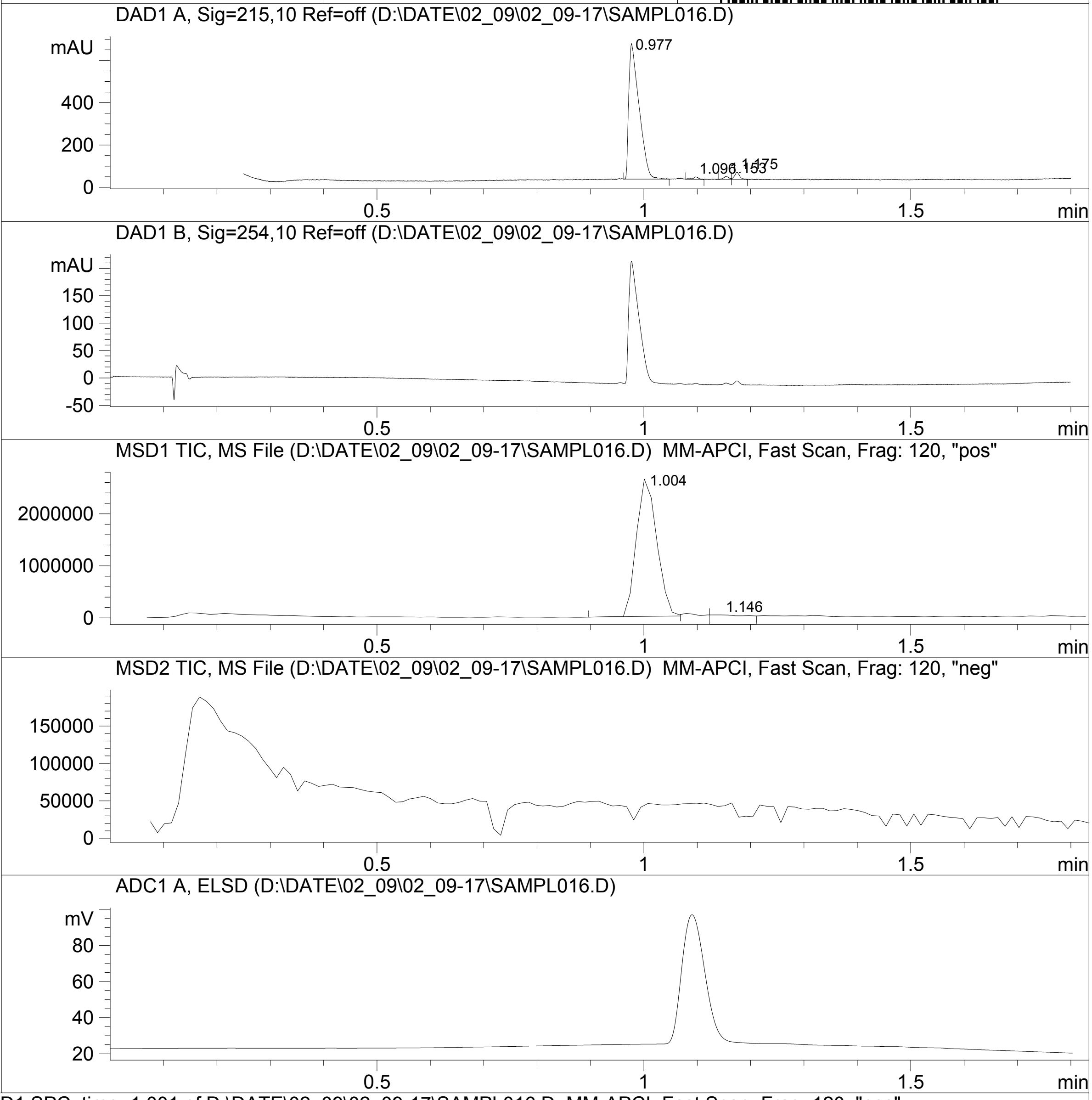

Mol Wt 471.571
Exact Mass 471.19

#	Time	Area%
1	0.977	95.13
2	1.096	1.15
3	1.153	1.15
4	1.175	2.56

RT 1.004

RT 1.146

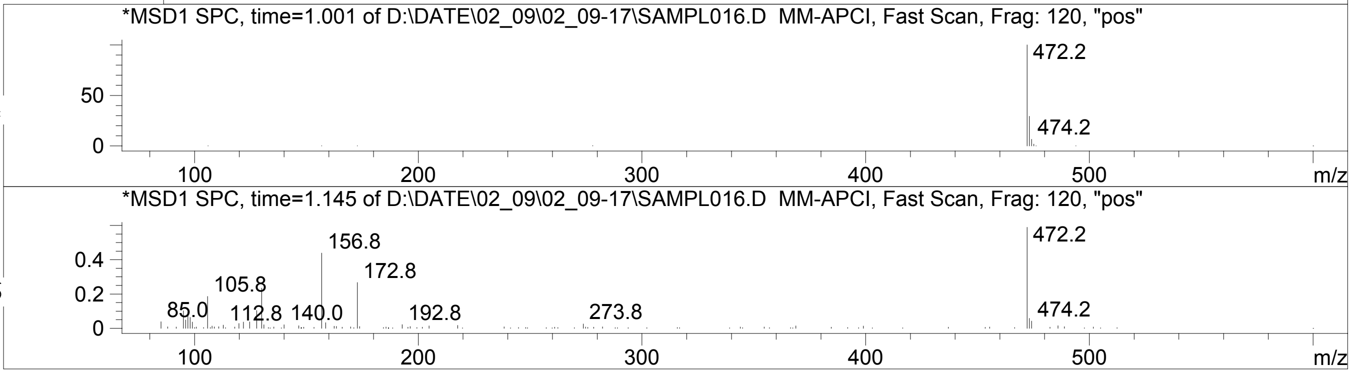

Compound ID: 7

Inj.Date 2/10/2011 O P2-F-02 RR Acq. Method C:\CHEM32\-> ->

MaxPeak: 93.22%
Ret_Time: 1.364 min

4532129

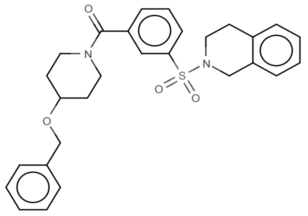

**Mol Wt**	**490.614**
**Exact Mass**	**490.23**

#	Time	Area%
1	1.078	3.92
2	1.247	1.58
3	1.309	1.28
4	1.364	93.22

DAD1 A, Sig=215,10 Ref=off (D:\DATE\01_11\01_10_14\SAMPL038.D)

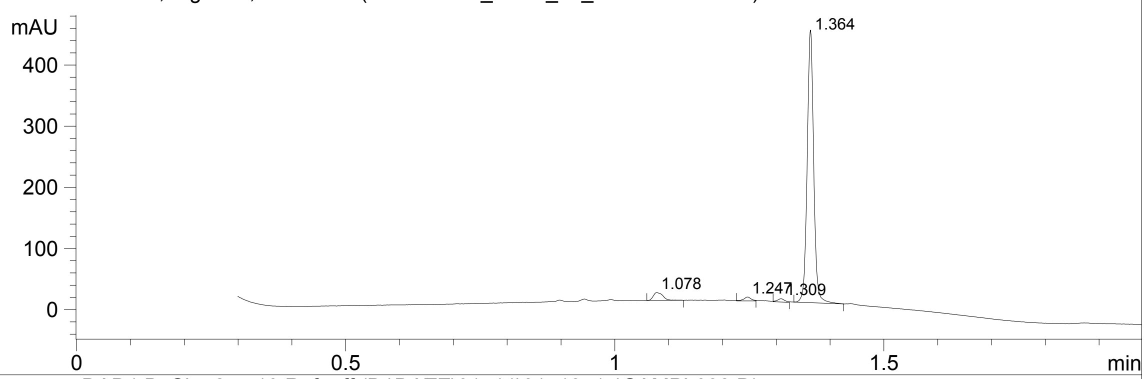

DAD1 B, Sig=254,10 Ref=off (D:\DATE\01_11\01_10_14\SAMPL038.D)

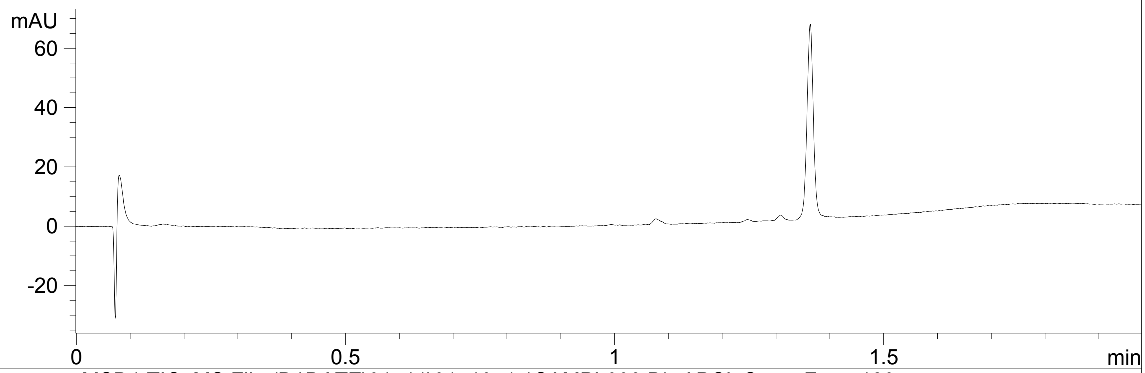

MSD1 TIC, MS File (D:\DATE\01_11\01_10_14\SAMPL038.D) APCI, Scan, Frag: 120

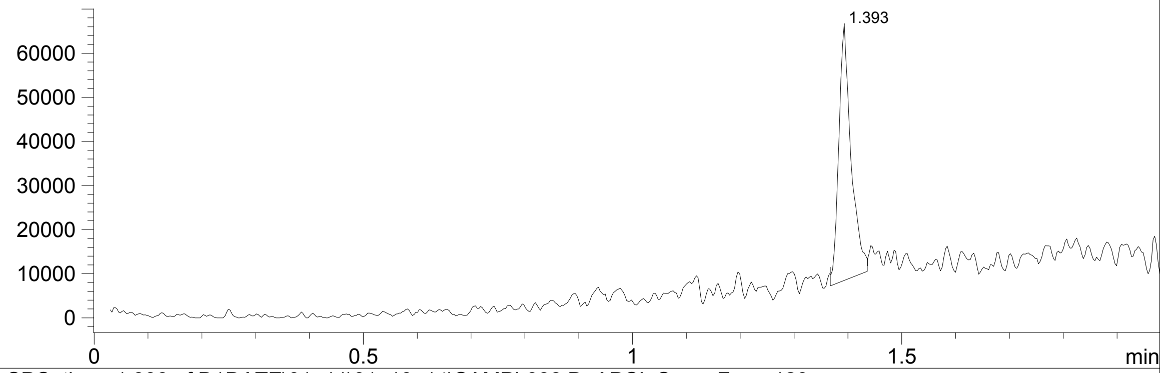

RT 1.393

*MSD1 SPC, time=1.393 of D:\DATE\01_11\01_10_14\SAMPL038.D APCI, Scan, Frag: 120

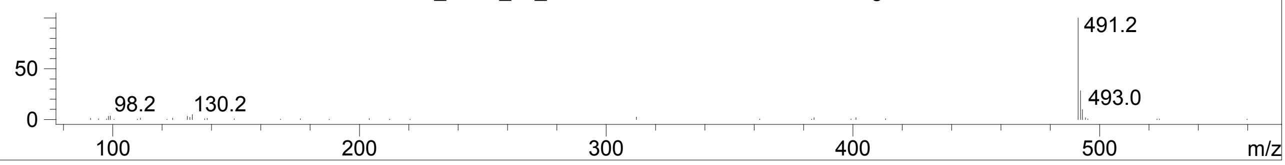

Compound ID: 8

Inj.Date 1/11/2011 L P2-E-01 VL Acq. Method C:\HPCHEM\-> ->

MaxPeak: 100.00%
Ret_Time: 1.306 min

4532153

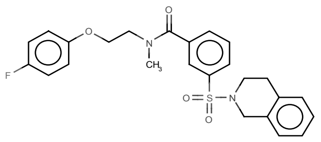

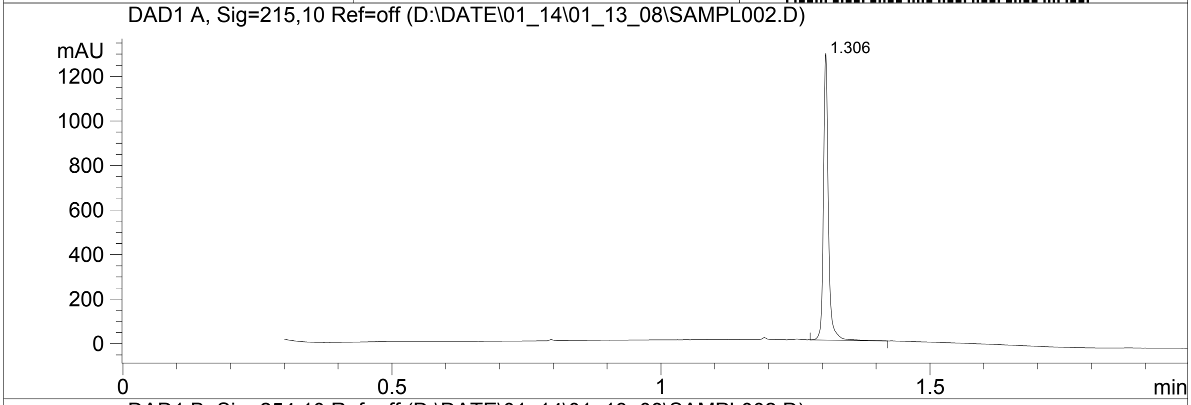

Mol Wt 468.54
Exact Mass 468.18

#	Time	Area%
1	1.306	100.00

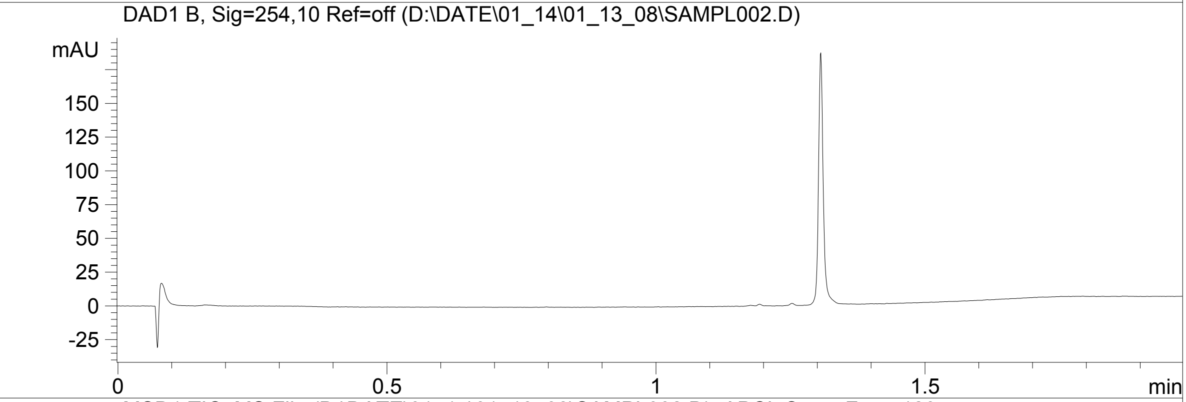

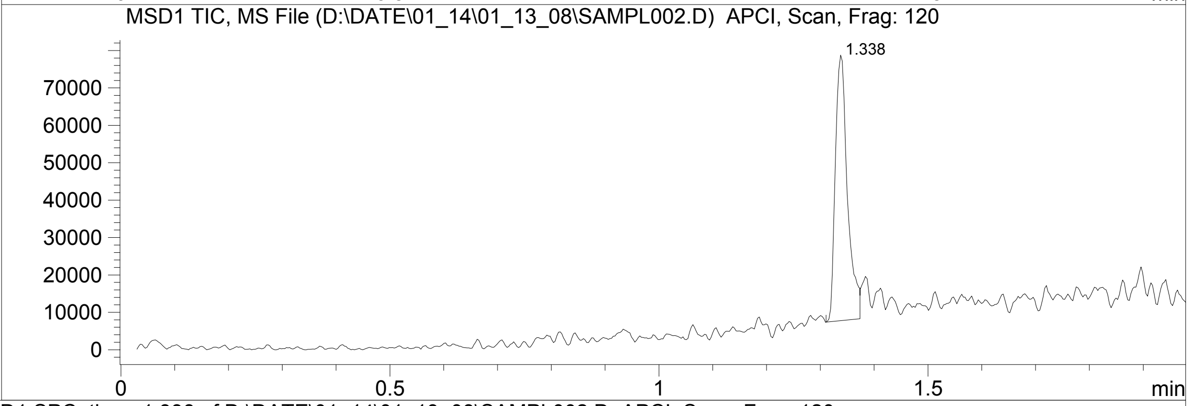

RT 1.338

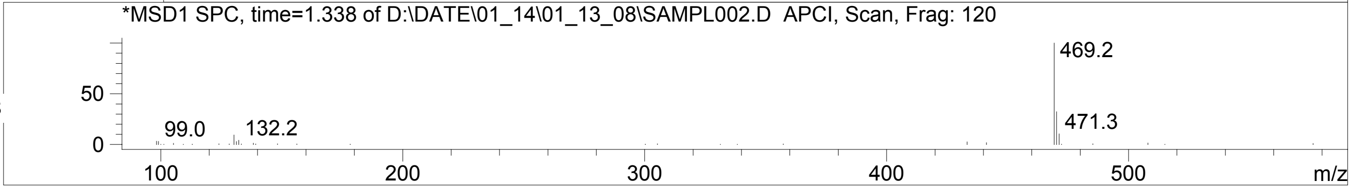

Compound ID: 9

Inj.Date 1/14/2011 L P2-A-01 VL Acq. Method C:\HPCHEM\-> ->

MaxPeak: 97.97%
Ret_Time: 1.158 min

4532004

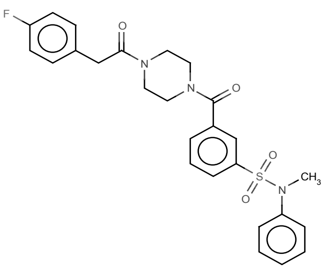

Mol Wt 495.566
Exact Mass 495.19

#	Time	Area%
1	0.718	2.03
2	1.158	97.97

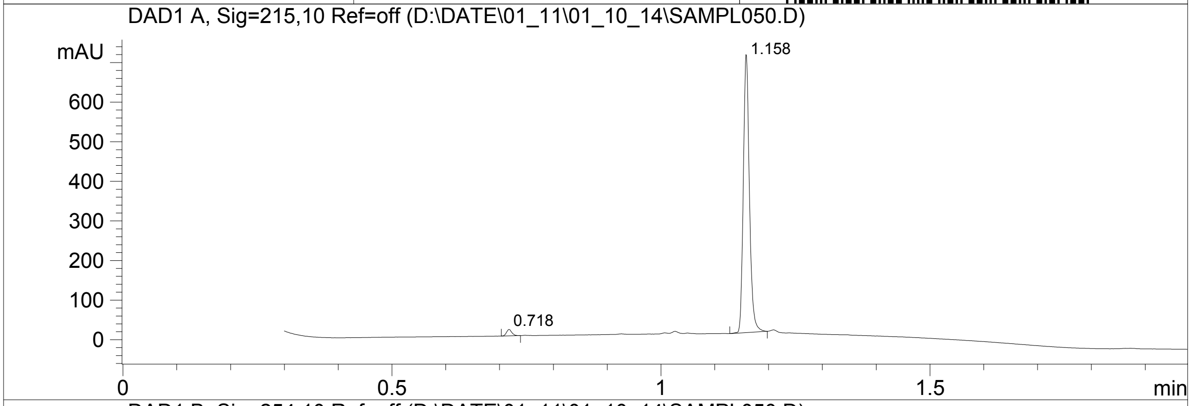

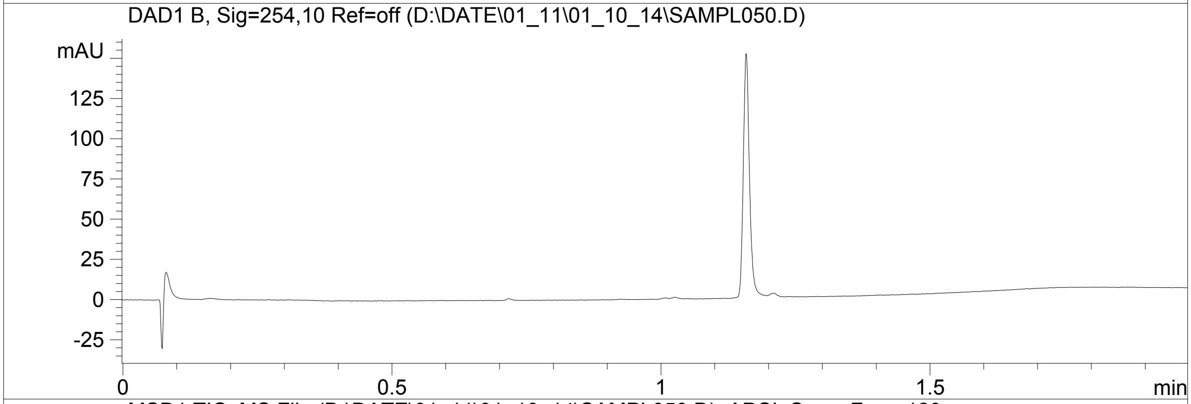

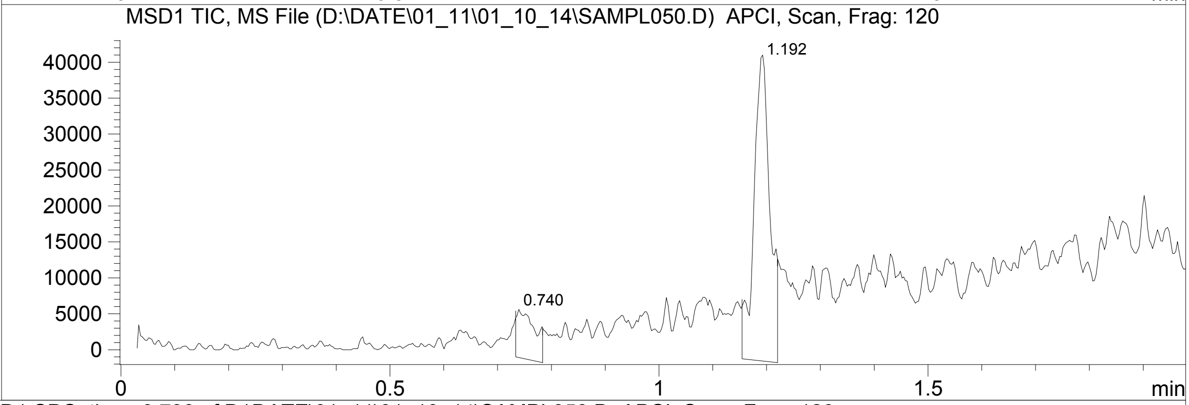

RT 0.740

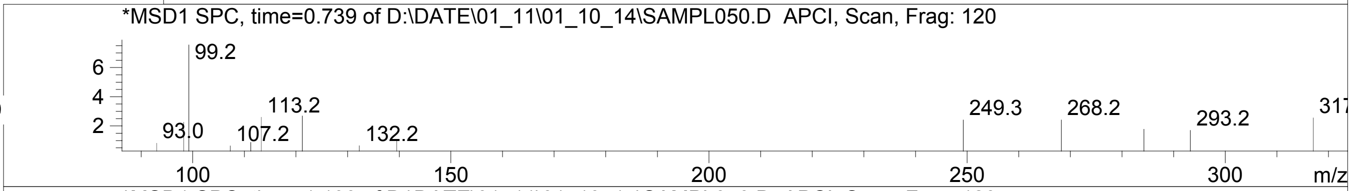

RT 1.192

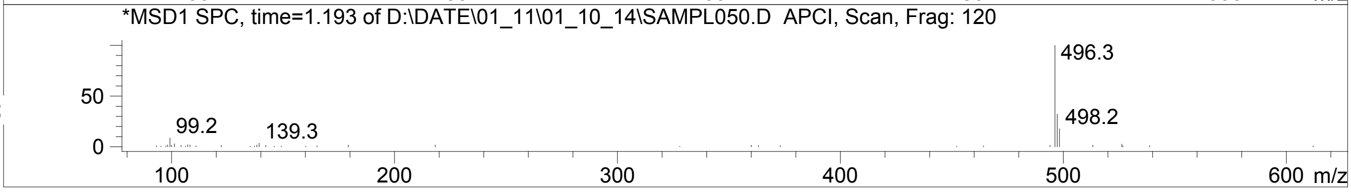

Compound ID: 10

Inj.Date 1/11/2011 L P2-F-04 VL Acq. Method C:\HPCHEM\-> ->

Sample Name: 1026936 OK

MaxPeak: 97.65%
Ret_Time: 0.731 min

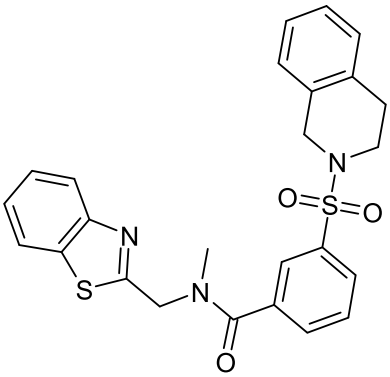

mw = 477,61

#	Time	Area%
1	0.696	2.35
2	0.731	97.65

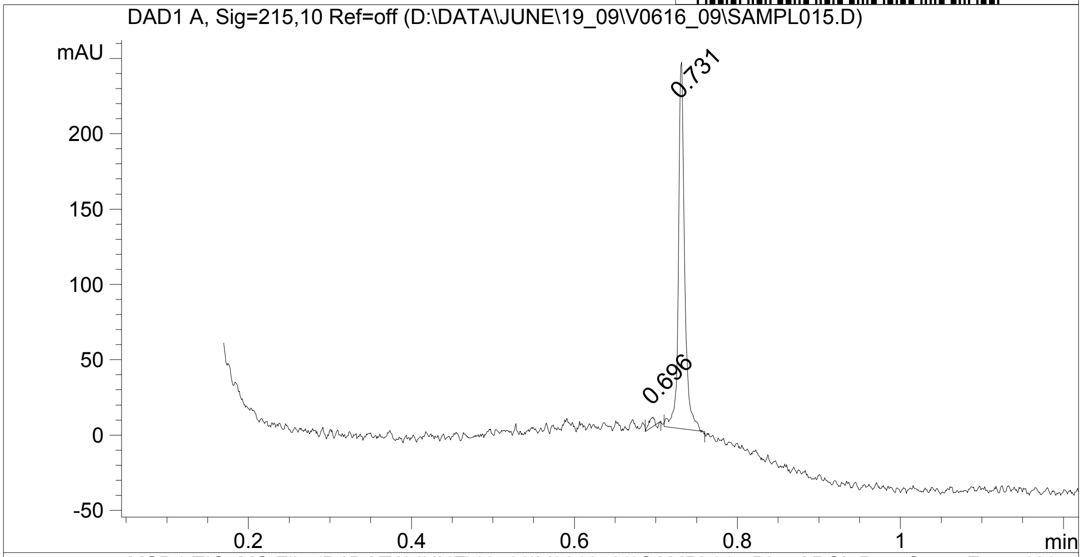

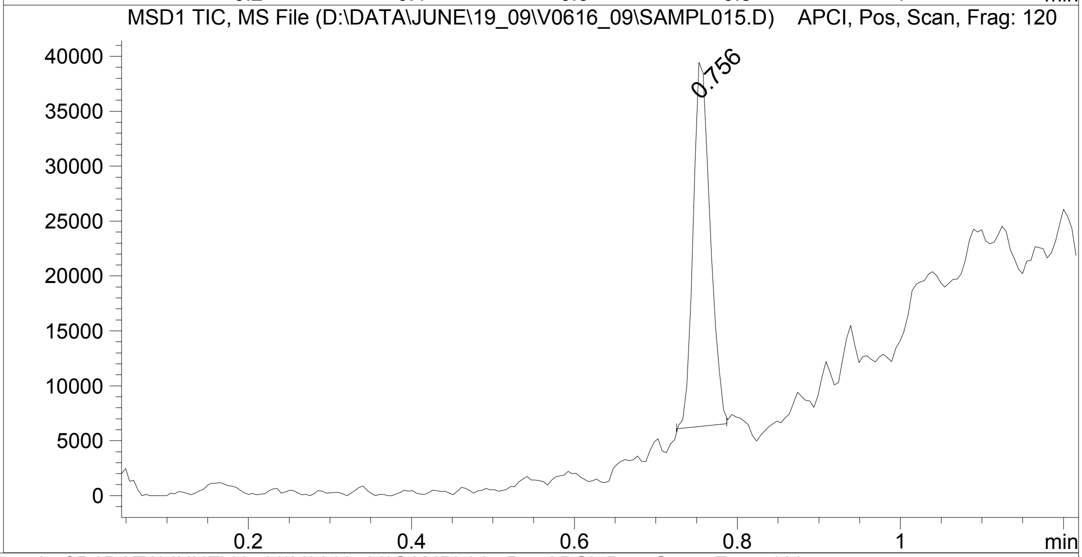

RT 0.756

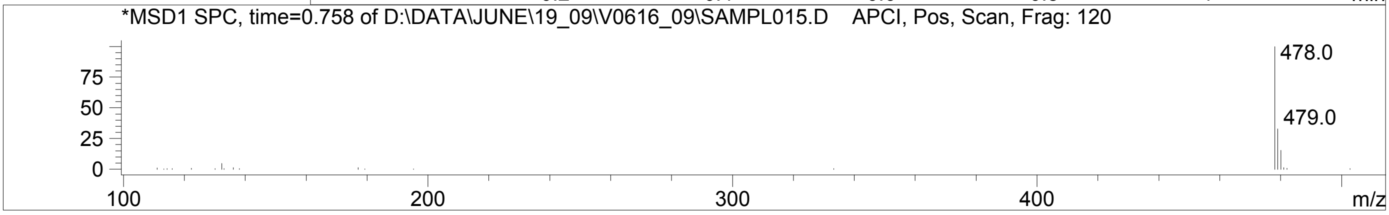

Compound ID: 11

Inj.Date 17.06.2006 L P2-B-08 Acq. Method SB15TFA3.M

MaxPeak: 96.68%
Ret_Time: 1.317 min

4532022

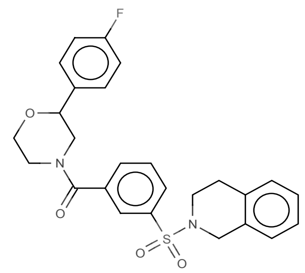

Mol Wt 480.551
Exact Mass 480.18

#	Time	Area%
1	0.752	1.71
2	1.079	1.61
3	1.317	96.68

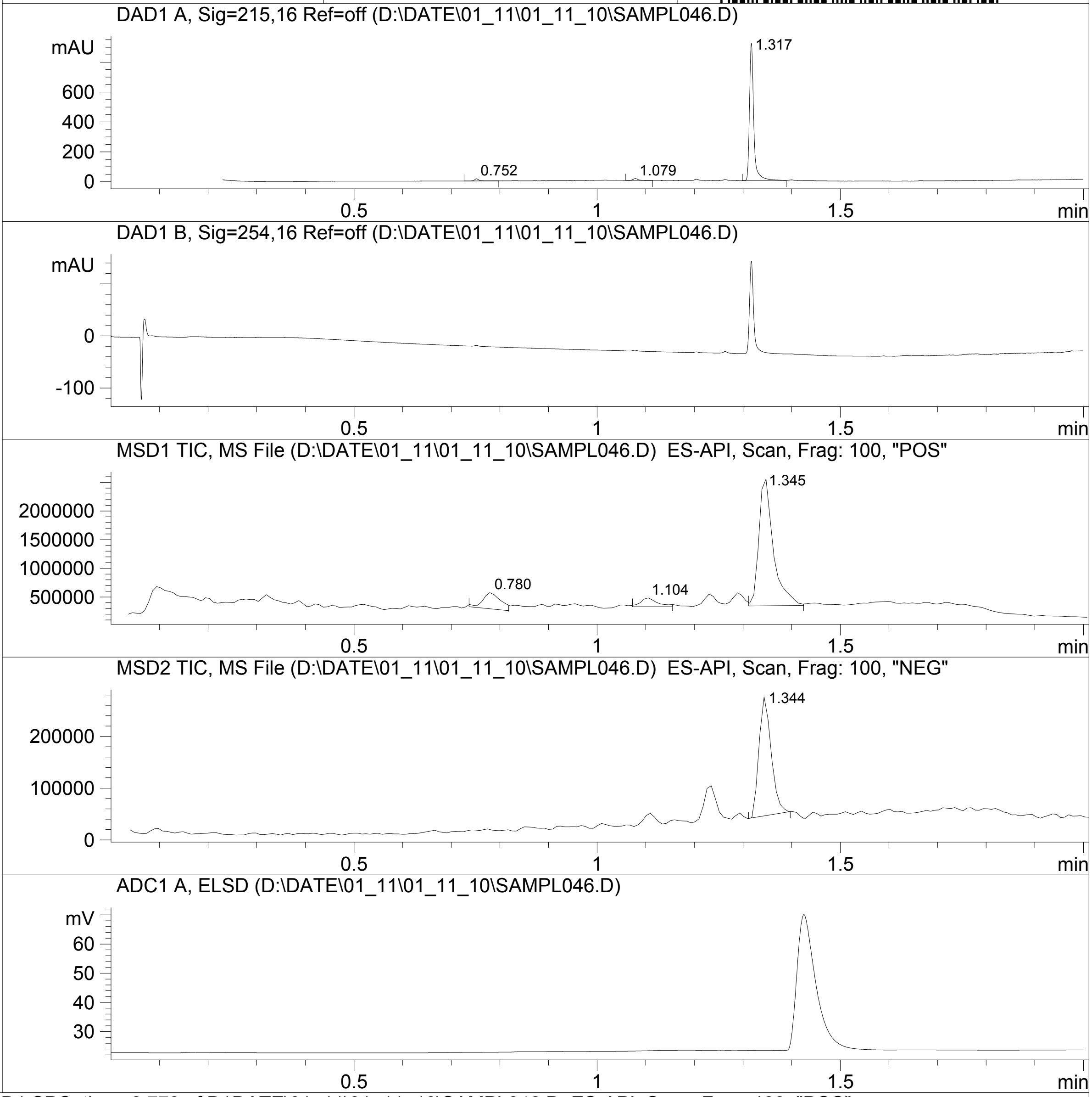

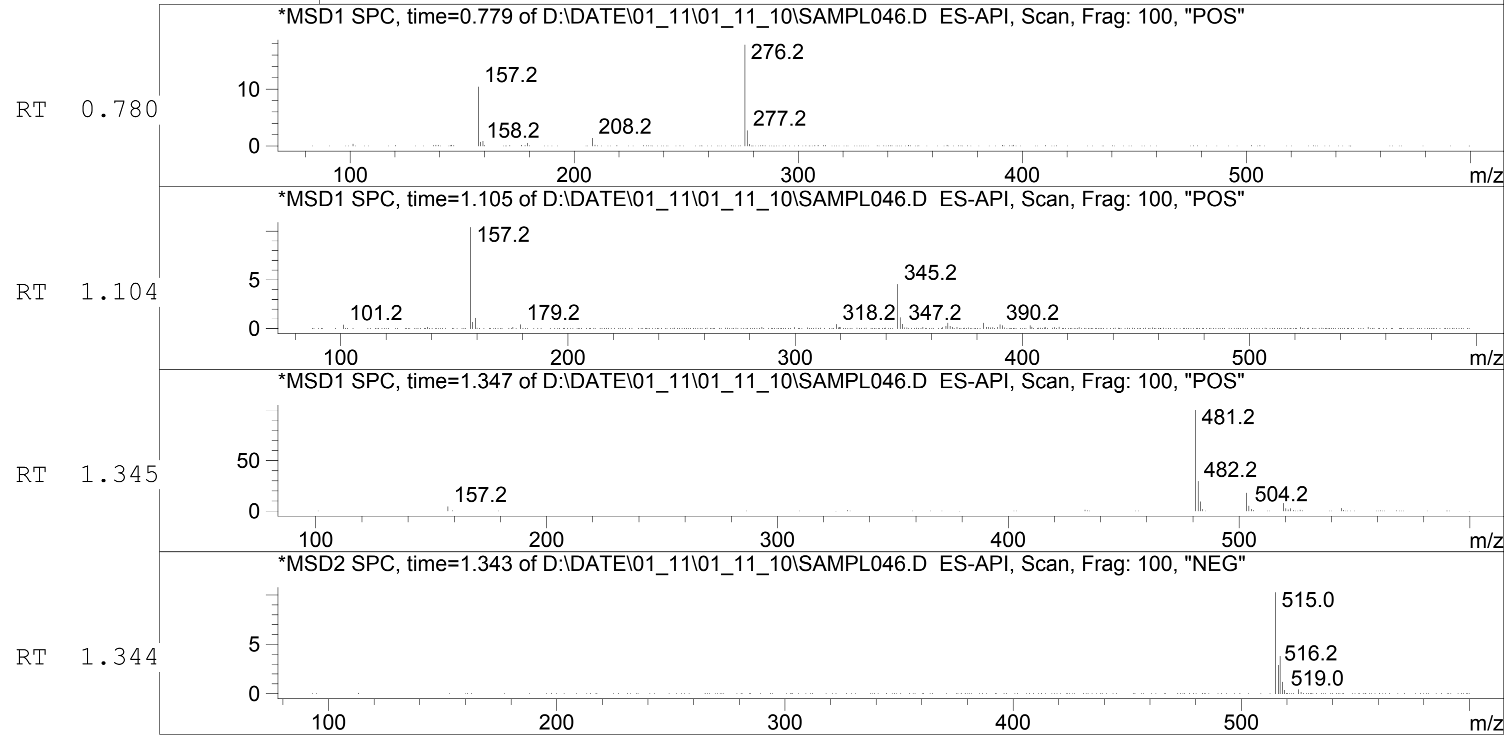

Compound ID: 12

Inj.Date 1/12/2011 O P2-E-09 '4' Acq. Method C:\CHEM32\-> ->

MaxPeak: 94.66%
Ret_Time: 1.240 min

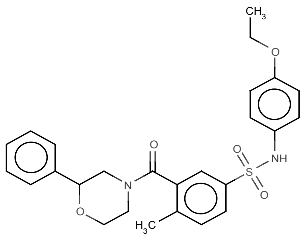

Mol Wt 480.576
Exact Mass 480.2

#	Time	Area%
1	1.009	1.14
2	1.240	94.66
3	1.267	4.19

4532001

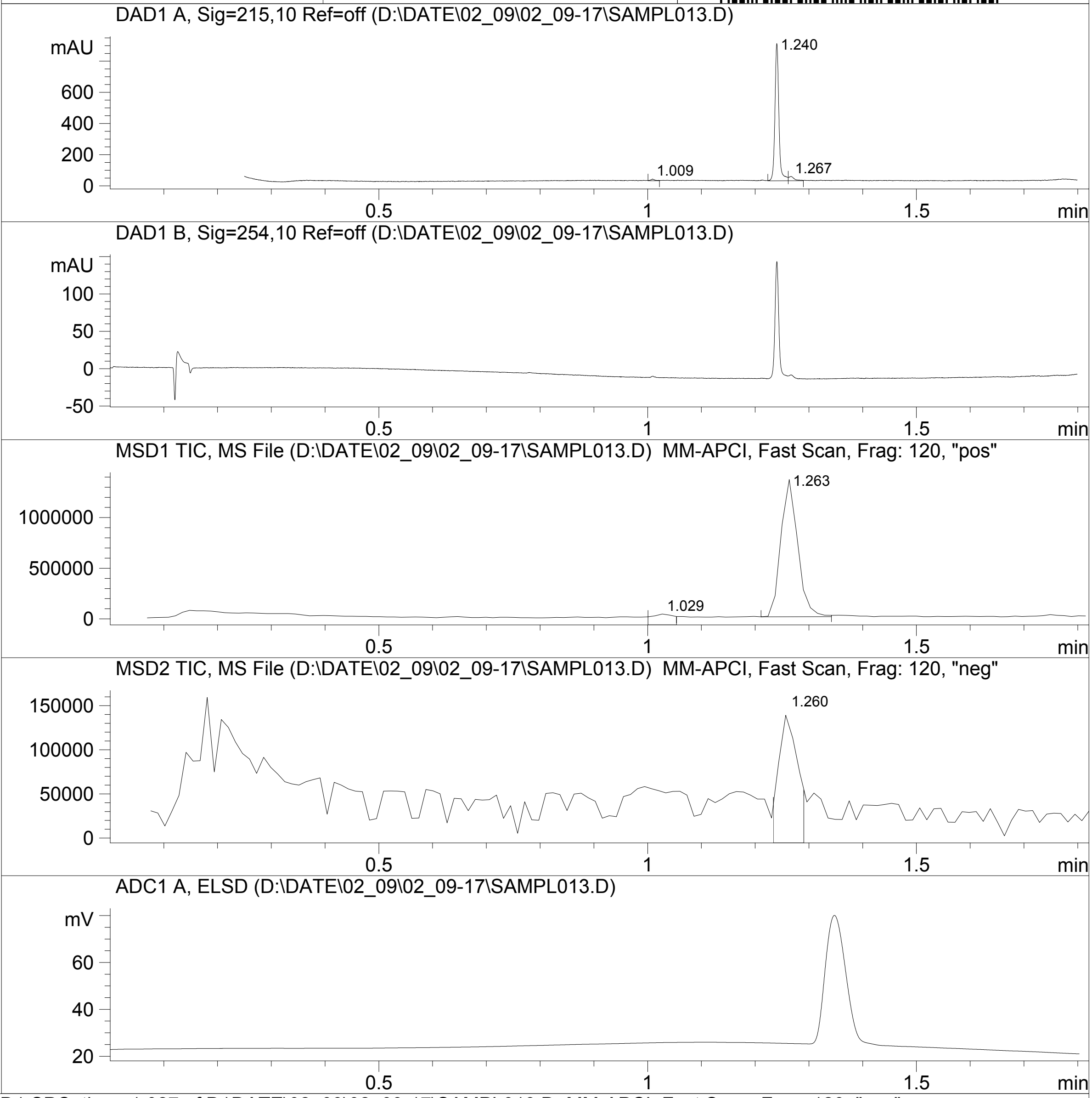

RT 1.029

RT 1.263

RT 1.260

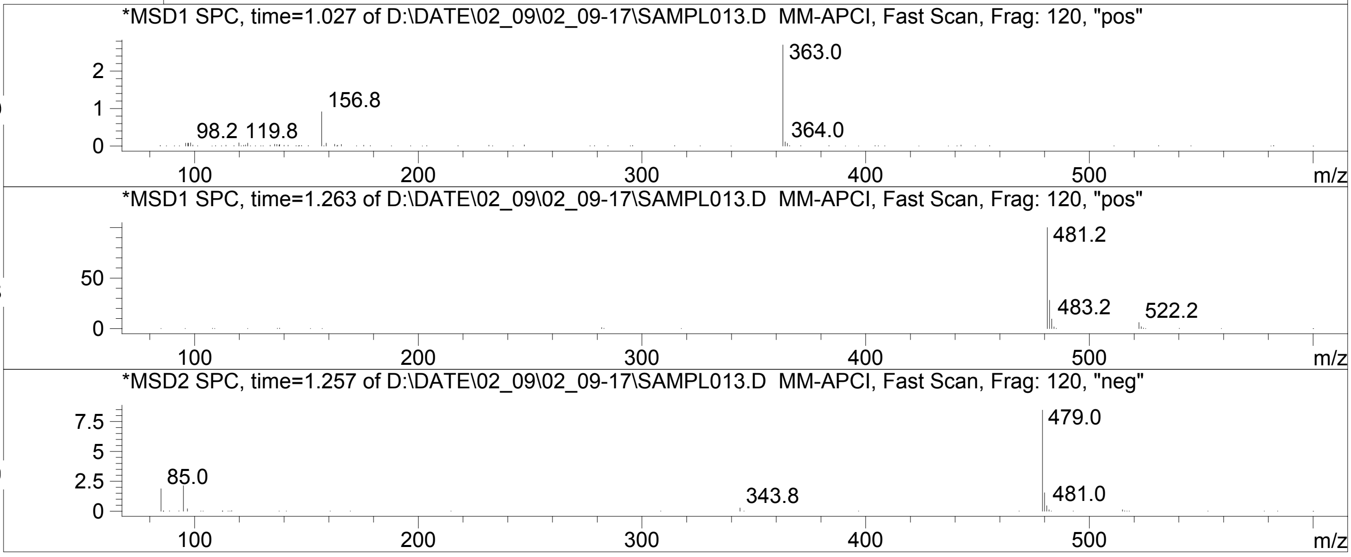

Compound ID: 13

Inj.Date 2/10/2011 O P2-E-08 RR Acq. Method C:\CHEM32\-> ->

MaxPeak: 100.00%
Ret_Time: 0.888 min

4532099

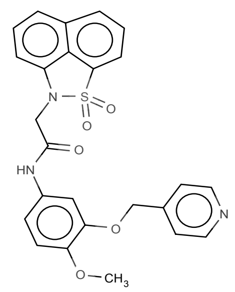

Mol Wt 475.516
Exact Mass 475.13

#	Time	Area%
1	0.888	100.00

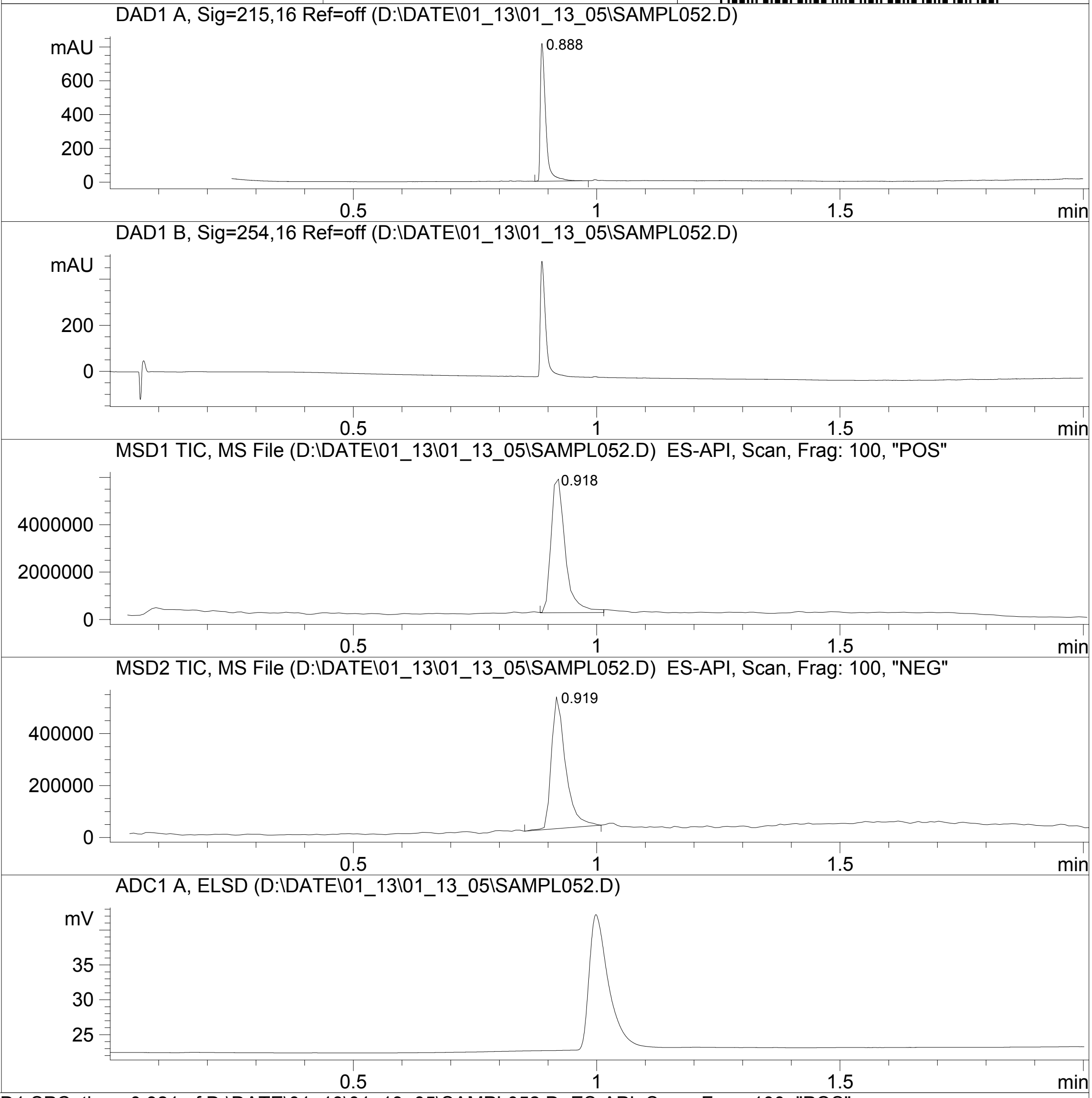

RT 0.918

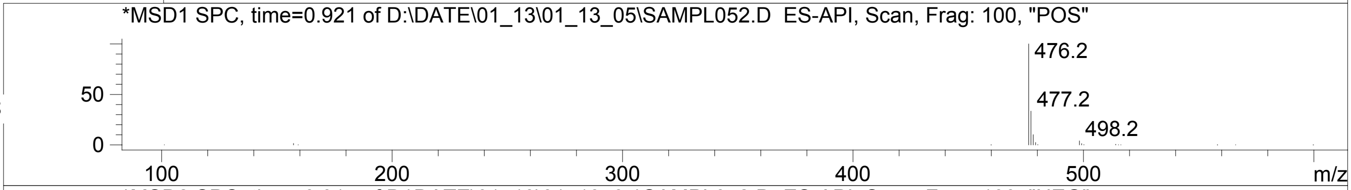

RT 0.919

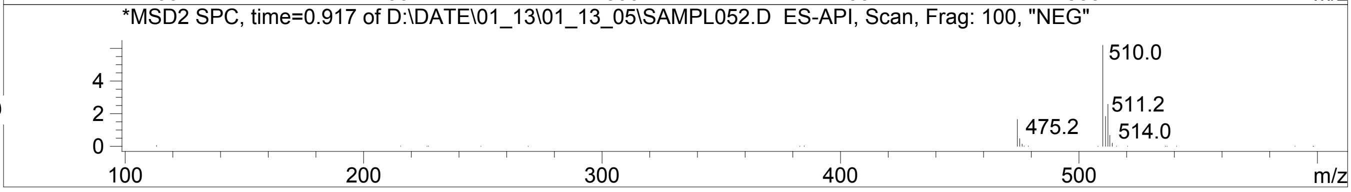

Compound ID: 14

Inj.Date 1/13/2011 O P2-F-06 '4' Acq. Method C:\CHEM32\-> ->
